# Influence of Fe and Mn on the Microstructure Formation in 5xxx Alloys—Part I: Evolution of Primary and Secondary Phases

**DOI:** 10.3390/ma14123204

**Published:** 2021-06-10

**Authors:** Jakob Grasserbauer, Irmgard Weißensteiner, Georg Falkinger, Thomas M. Kremmer, Peter J. Uggowitzer, Stefan Pogatscher

**Affiliations:** 1 Christian Doppler Laboratory for Advanced Aluminum Alloys, Chair of Nonferrous Metallurgy, Montanuniversitaet Leoben, Franz-Josef Straße 18, 8700 Leoben, Austria; irmgard.weissensteiner@unileoben.ac.at (I.W.); stefan.pogatscher@unileoben.ac.at (S.P.); 2 AMAG Rolling GmbH, 5282 Ranshofen, Austria; georg.falkinger@amag.at; 3 Chair of Nonferrous Metallurgy, Department Metallurgy, Montanuniversitaet Leoben, Franz-Josef Straße 18, 8700 Leoben, Austria; thomas.kremmer@unileoben.ac.at (T.M.K.); peter.uggowitzer@mat.ethz.ch (P.J.U.)

**Keywords:** aluminum alloys, AlMg(Mn), solidification, constituents, dispersoids, cold rolling, microstructure

## Abstract

The increasing demands for Al sheets with superior mechanical properties and excellent formability require a profound knowledge of the microstructure and texture evolution in the course of their production. The present study gives a comprehensive overview on the primary- and secondary phase formation in AlMg(Mn) alloys with varying Fe and Mn additions, including variations in processing parameters such as solidification conditions, homogenization temperature, and degree of cold rolling. Higher Fe alloying levels increase the primary phase fraction and favor the needle-shaped morphology of the constituent phases. Increasing Mn additions alter both the shape and composition of the primary phase particles, but also promote the formation of dispersoids as secondary phases. The size, morphology, and composition of primary and secondary phases is further affected by the processing parameters. The average dispersoid size increases significantly with higher homogenization temperature and large primary particles tend to fragment during cold rolling. The microstructures of the final soft annealed states reflect the important effects of the primary and secondary phase particles on their evolution. The results presented in this paper regarding the relevant secondary phases provide the basis for an in-depth discussion of the mechanisms underlying the microstructure formation, such as Zener pinning, particle stimulated nucleation, and texture evolution, which is presented in Part II of this study.

## 1. Introduction

In the past decades, the use of aluminum as a construction material strongly increased in various fields of application. Depending on the alloying and micro-alloying elements, a wide range of combinations of materials properties exist. Especially with regard to improving sustainability and reducing CO_2_ emissions of processes and technical applications, the use of aluminum alloys as replacement for materials with higher density is a common approach. With the growing importance of weight reductions in the automotive sectors, amongst other alloying systems, attention was focused on the aluminum 5xxx (AlMg(Mn)) alloys. The non-age-hardenable alloys typically show superb combinations of medium strength, corrosion resistance, and good formability [1]. Control of the mechanical properties is predominantly realized by the amount of Mg in solid solution and the degree of cold work prior to soft annealing [1]. Additionally, the secondary alloying element Mn plays a key role in intermetallic phase formation. It therefore affects both the processing and final properties of the alloys.

The importance of primary phase distribution in the AlMg(Mn) alloys is highlighted by their capability of reducing the Lüders elongation and the resulting undesired strain marks, which are a common problem of those alloys [2]. In this context, the industrial casting process is crucial as the different cooling rates in ingot casting, continuous casting, and twin-roll casting strongly influence the primary phase size and number density [1]. Overall, the development of advanced aluminum alloys hence requests both a sophisticated process design and deep knowledge on microstructural relations in these alloys [1,3,4,5].

The primary and secondary phase formation in 5xxx Al alloys is in general a very complex process and controlled by Mg and secondary alloying elements such as Mn, Fe, and Si [6]. Although a large variety of intermetallic phases can form during solidification, and also during subsequent heat-treatments, the most typically found phases include: Al_3_Fe (or Al_13_Fe_4_), Al_6_Mn, (α-)Al(Fe,Mn)Si, and Mg_2_Si [6,7,8,9,10,11,12,13,14,15,16,17,18].

The Fe-rich intermetallic Al_3_Fe tends to form in alloys showing higher Fe/Mn ratios and lower amounts of Si or Mg. This phase, appearing needle-like in 2D micrographs, is highly implicated with disadvantageous effects of Fe containing intermetallics in Al. Because of their characteristic shape, those precipitates cause stress concentrations and, additionally, the phase is reported to negatively influence the corrosion resistance of the alloys [14,19,20]. According to thermodynamic calculations and experimental observations, the Al_3_Fe phase is often found as Al_13_Fe_4_. Because of the interchangeability of Fe and Mn especially at lower ratios of Fe/Mn and higher temperatures, Al_3_(Fe,Mn) or Al_13_(Fe,Mn)_4_ is recommended as a more precise designation [6,9,10,16]. In the following we stick to this notation.

The intermetallic phase Al_6_Mn, well known from the group of 3xxx (AlMn) alloys, shows a stronger formation with low Fe/Mn ratio and low Si contents. Due to the interchangeability of Fe and Mn, the Al_6_(Fe,Mn) phase is formed which mostly appears in the form of irregularly shaped plate- or block-like particles, but sometimes also exhibits Chinese-script structure [16]. The exact stoichiometry of those phases in the Mg-containing 5xxx alloys is hence not totally clarified, e.g., the authors of [9,21] report on the formation of Al_m_(Fe,Mn) with m being in the range of 4.0 to 4.4. They additionally mention the influence of higher Mg content in promoting the formation of Al_3_(Fe,Mn) at the expense of Al_6_(Fe,Mn) [9,12,21].

Regarding the influence of Si on the formation of Fe and Mn containing phases in Al alloys, the two different types of α- and β-AlFeSi must be considered. In the case of lower amounts of Fe impurities and the absence of Mn especially in AlMgSi alloys, typically β-Al_5_FeSi formation is favored. This generally faceted and needle-shaped phase shows similar effects on the alloys mechanical properties as the Al_3_(Fe,Mn). The tendency of formation or transformation from β- to α-AlFeSi is promoted by higher Fe/Si ratios and requires a minimum amount of Mn for phase stabilization. While the hexagonal α_h_-Al_8_FeSi is more often found in Mn free alloys, cubic α_c_-Al_15_(Fe,Mn)_3_Si_2_ is formed by peritectic reactions and is mostly affected by the Mn content. In contrast to β-Al_5_FeSi, their globular or Chinese-script morphology can reduce the adverse effects of the intermetallic particles on the mechanical properties [6,7,8,11,15,22,23].

In addition to the Fe and Mn bearing primary phases, Mg_2_Si particles can form during solidification as an equilibrium phase. The phase fraction of these particles largely depends on Si and Mg contents as well as on the cooling rates. During subsequent heat-treatments, those Mg_2_Si precipitates can be dissolved and/or modified in size and shape to meet their intended purpose as hardening phases in (especially 6xxx) Al alloys [1,6].

The cooling rates during solidification are crucial for the phase formation in AlMg(Mn) alloys. Distinguishing between slow cooling (S-C, ~0.5–3 K/s) and near-rapid cooling (NR-C, ~50 K/s), the stabilities of the aforementioned phases can change considerably [12]. Moreover, the network of intermetallic phases is significantly refined with higher cooling rates. Furthermore, the amount of solutes of especially Fe, Mn, and Si is affected by the solidification rate, which in turn plays a decisive role in the subsequent homogenization treatment and the formation of secondary phase particles [7,12,14,16,23].

Depending on the particular time and temperature of the heat-treatments in Fe and Mn containing 5xxx Al alloys, three different types of particles are involved in the precipitation behavior of secondary phases: Al_6_(Fe,Mn), α-Al_15_(Fe,Mn)_3_Si_2_, and Mg_2_Si. The rod- or platelet-shaped Al_6_(Fe,Mn) dispersoids are widely used as pinning particles in those alloys and therefore have significant influence on the recrystallization behavior and the microstructure. Different studies highlight the importance of maximum homogenization temperature and holding time on the volume fraction and size distribution of the particles [24,25,26], indicating that higher temperatures result in a higher fraction of coarsened Al_6_(Fe,Mn) particles for example. Additionally, the higher temperatures affect the level of solute Si as Mg_2_Si starts to dissolve at temperatures above 500 °C, and β- to α-AlFeSi transformations of primary phases can occur [10,22]. As a consequence, the likelihood of α-Al_15_(Fe,Mn)_3_Si_2_ dispersoid formation increases. The nucleation and growth of those particles is interdependent of the Al_6_(Fe,Mn) dispersoid formation, which highlights the importance of well-considered thermomechanical processing for these Al alloys [24,25,26,27,28,29].

In recent decades, the usage of thermodynamic calculations was established in alloy design. With different commercially distributed software packages (e.g., FactSage, Thermo-Calc, MatCalc, and Pandat), the design of novel alloy compositions or modified thermomechanical processing is more easily accessible. Calculations of equilibrium and non-equilibrium conditions allow careful predictions of the occurring phases, although the validation by experimental characterizations is still indispensable [8,10,26].

The present study investigates the influence of secondary alloying elements Fe and Mn in a near 5182 aluminum alloy produced in laboratory scale. Attention is paid to primary and secondary phase formation in dependence on alloy composition and cooling rate during solidification as well as precipitation and phase transformation during homogenization. Additionally, a comparison to thermodynamic calculations highlights the importance of combining experimental work with simulation studies.

## 2. Materials and Methods

The present study involved four modifications of Fe and Mn contents on a Al4.5Mg alloy produced within a laboratory scaled process. The compositions of the alloys can be found in Table 1.

The laboratory sample production followed the manufacturing process of 5xxx Al alloys as described in [30,31]. Casting at two different scales resulted in two different cooling rates during solidification to approximately simulate the cooling rates in twin-roll casting (with laboratory near rapid cooling, NR-C) and continuous casting (with laboratory slow cooling, S-C).

After milling of the cast blocks to sizes of 60 × 40 × 14 mm^3^, two different homogenization treatments were performed (maximum temperatures of 500 and 550 °C). The heat treatment cycle, using an air-circulated furnace (Naberthem N60/85SHA, Lilienthal, Germany), included heating from room temperature within five hours to the different maximum temperatures and holding for two hours.

The following rolling process was performed on a laboratory rolling mill with non-heatable rolls measuring 80 mm in diameter. For hot rolling (HR), the samples were first heated to the rolling temperature of 430 °C in the air-circulated furnace and put back into it after each rolling step for about 10 min to avoid critical losses in temperature. The final hot rolling thickness was varied to allow different degrees of cold rolling (CRD) while keeping the final sheet thickness unchanged. Therefore, the hot rolling process comprises reductions from 14 to 3.20 mm as well as 14 to 1.85 mm. The following intermediate annealing with a maximum temperature of 370 °C and a total cycle-time of about 72 h was again done in the air-circulated furnace. Subsequent cold rolling at room temperature was carried out on the aforementioned rolling mill. Reaching the final sheet thickness of about 1.20 mm resulted in two different cold rolling degrees (CRD) of 35% and 63%, respectively.

The final soft annealing was performed in a salt bath, which provides faster heating than conventional air-circulated furnaces. The annealing time was 5 min at a temperature of 500 °C, after which the samples were quenched in water.

Metallographic sample preparation for microstructure analyses comprised gentle grinding and polishing with diamond and oxide polishing suspensions. Preparation for transmission electron microscopy was finished by electropolishing of discs having 3 mm in diameter and approximately 100 µm in thickness. An electrolyte composing of ⅓ HNO_3_ and ⅔ methanol was used in a Struers TenuPol twin-jet electropolishing unit at temperatures around −25 °C.

The as-cast homogenized and soft annealed sample states were investigated using a scanning electron microscope (SEM) (JEOL 7200F FEG-SEM, Tokyo, Japan) equipped with an energy dispersive X-ray (EDX) detector (XMax-80, Oxford Instruments, Abingdon, UK) including automatic particle feature analysis of backscattered electron images using threshold limits. The feature analysis was carried out for as-cast and homogenized samples containing at least 1000 particles per scan for statistically representative results. For characterization Fe/Mn-ratios as well as shape factor ($\mathrm{SF}=\frac{Perimeter^{2}}{4\pi *Area}$) were used directly from feature mapping data. Focus was placed on primary phase distribution and composition in the as cast and homogenized sample states as well as the distribution and composition of dispersoids in the homogenized samples. Additionally, transmission electron microscopy (Thermo ScientificTM TalosTM F200X G2, ThermoFisher Scientific, Hillsboro, OR, USA) was used to clarify the composition of the second phase particles.

The evaluation of the primary and secondary phase fraction was carried out with the help of the free software tool ImageJ (version 1.53e). The calculations of the dispersoid volume fractions followed the method of Österreicher et al. [32], analyzing SEM images of higher magnifications (10,000×; at least a total area of 325 µm^2^) recorded using an accelerating voltage of 5 kV. The results were recorded including the average radii (r) and aspect ratios (AR; shorter divided by longer aspect of the particles) of the particles. Note that the related tables display the average data of all evaluated images per sample, while the images of the individual samples shown in the respective figures may show slight deviations thereof.

The thermodynamic calculations for this study followed the CALPHAD approach using the integrated computational software tool Pandat, database PanAl2019 (CompuTherm LLC, Middleton, WI, USA). The simulation involved calculations for non-equilibrium as-cast (Scheil approach [33]) as well as equilibrium conditions. The results are discussed and evaluated by contrast with the experimental findings.

## 3. Results

This section presents the evolution of primary and secondary phases in the alloys described above using micrographs as well as phase fraction analysis. Additional thermodynamic calculations using Pandat are shown in comparison and for experimental validation. Primary and secondary phase fractions and composition were derived by the evaluation of multiple SEM pictures and feature (EDX) mappings.

### 3.1. Microstructures and Intermetallic Phases in As-Cast State

#### 3.1.1. Slow Cooling during Solidification (S-C)

**Figure 1:**
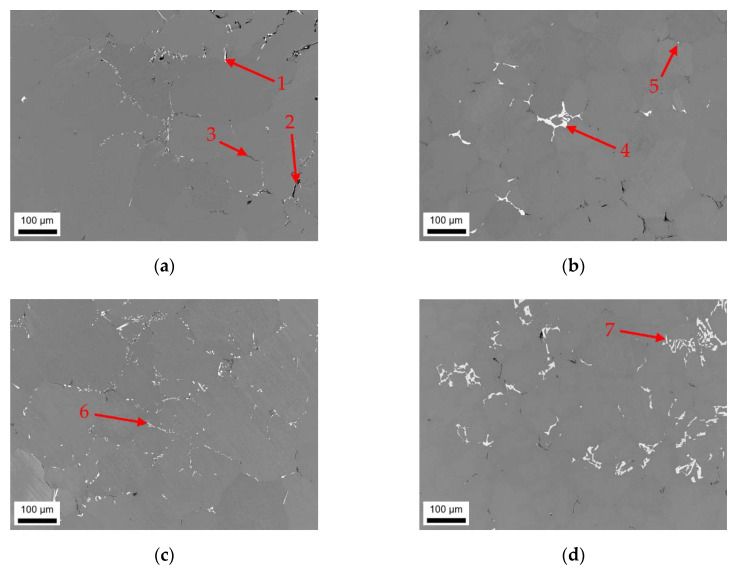
Casting microstructure of S-C cast samples; (**a**) LFe-LMn, (**b**) LFe-HMn, (**c**) HFe-LMn, and (**d**) HFe-HMn; (1) Al_3_(Fe,Mn) phase, (2) casting defect (pore), (3) Mg_2_Si phase, (4) Al_6_(Fe, Mn) phase, (5) Al_15_(Fe,Mn)_3_Si_2_ phase, (6) Al_3_(Fe,Mn) phase, (7) Chinese-script Al_6_(Fe,Mn) phase.

**Figure A1:**
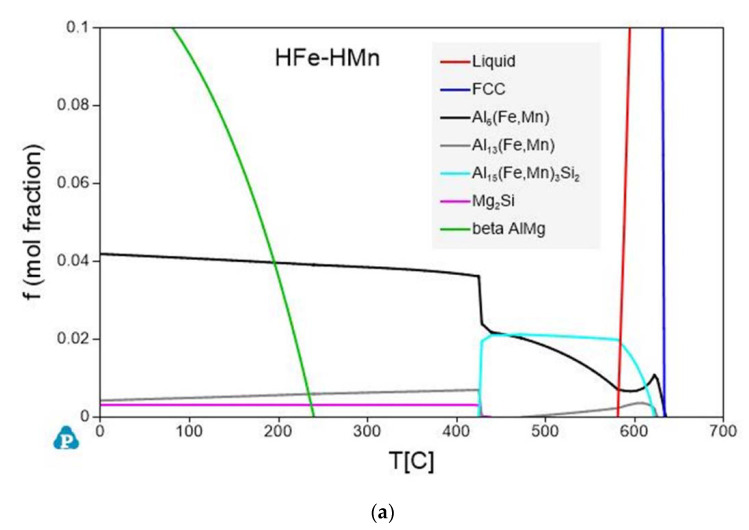

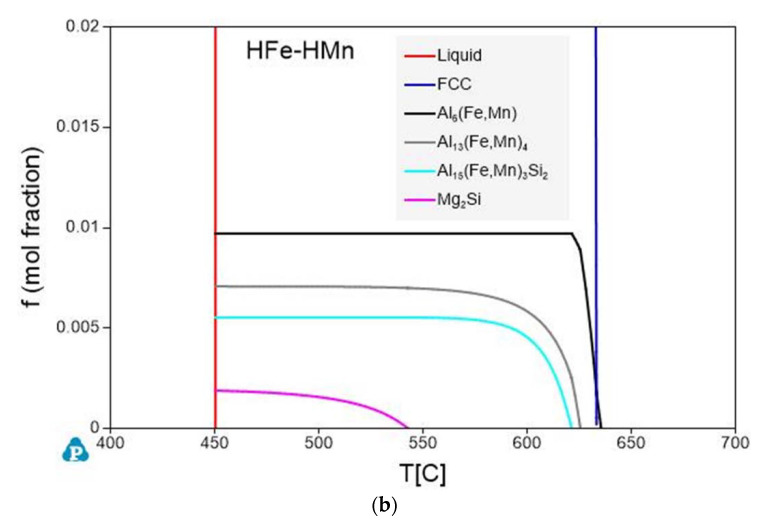
Thermodynamic simulation of the phase evolution during cooling for (**a**) nonequilibrium (Scheil) and (**b**) equilibrium solidification for the HFe-HMn alloy.

The as-cast microstructures of the four different alloys were analyzed in terms of particle size and distribution of the primary phases using SEM and EDX. Figure 1a–d illustrate sizes and arrangement of the microstructural features as well as the casting defects in the alloys according to Table 1. Depending on the alloy’s Fe and Mn contents, different primary phase structures can be observed, mainly segregated to the casting cell boundaries. To illustrate the phase evolution during cooling, a thermodynamic simulation for equilibrium and nonequilibrium solidification (Scheil) for the HFe-HMn alloy is given in Figure A1.

**Figure A2:**
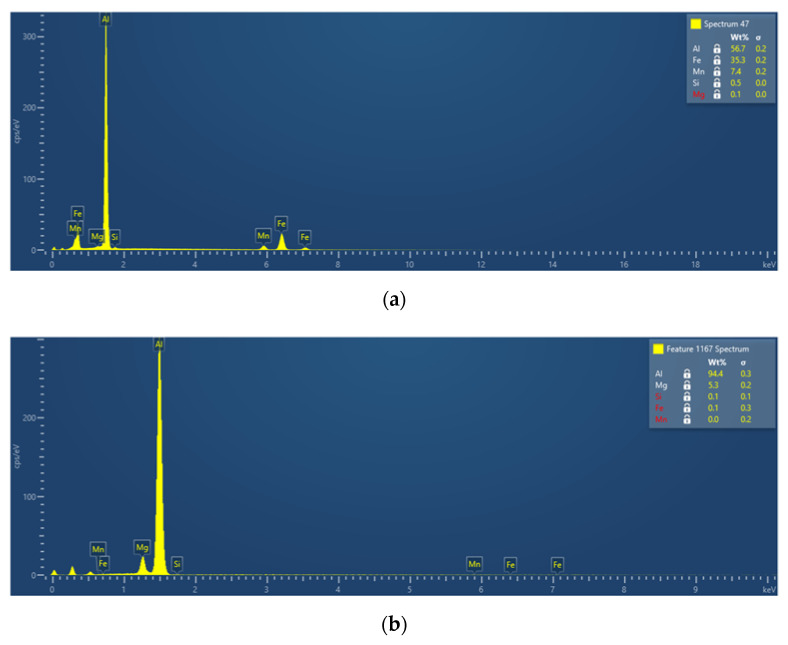

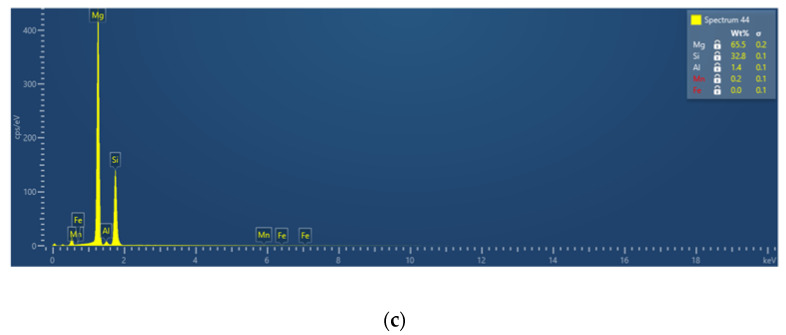
EDX point analysis spectra indicated by the marked spots in Figure 1; (**a**) Figure 1a (1), Al_3_(Fe,Mn) particle, (**b**) Figure 1a (2), pore, (**c**) Figure 1a (3), Mg_2_Si particle.

**Figure A3:**
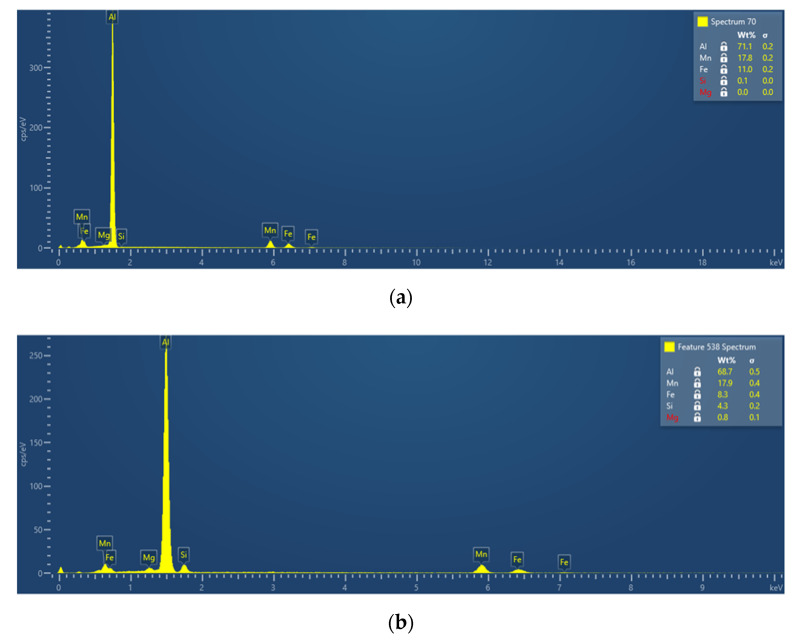
EDX point analysis spectra indicated by the marked spots in Figure 1; (**a**) Figure 1b (4), Al_6_(Fe,Mn) particle, (**b**) Figure 1b (5), Al_15_(Fe,Mn)_3_Si_2_ particle.

**Figure A4:**
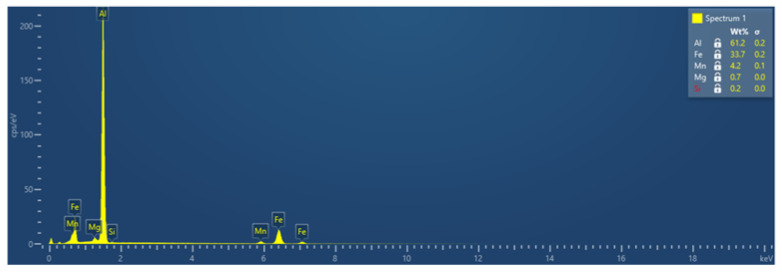
EDX point analysis spectra indicated by the marked spots in Figure 1c (6), Al_3_(Fe,Mn) particle.

**Figure A5:**
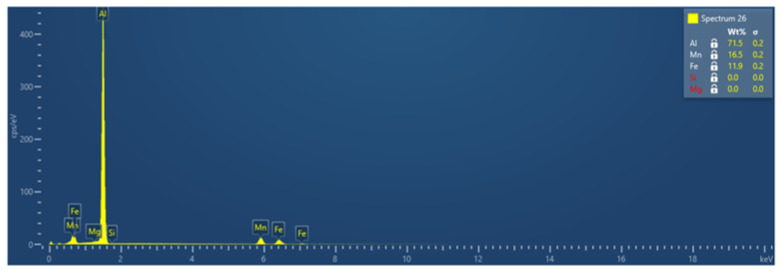
EDX point analysis spectra indicated by the marked spots in Figure 1d (7), Al_6_(Fe,Mn) particle.

In the samples with low Mn content (LFe-LMn and HFe-LMn) in Figure 1a–c, the size and density of the intermetallic network obviously differs. While LFe-LMn shows looser structured, typically needle-shaped bright phases, an increase in Fe content results in a higher number density and slight coarsening of those intermetallics. The characteristic shape of the phases (Figure 1a (1) and Figure 1c (6)) is not altered with the change in Fe contents. EDX analysis of the particles (features) indicates Al_3_(Fe,Mn) type for the phases (see Figure A2, Figure A3, Figure A4 and Figure A5 for the EDX spectra).

Additionally, Figure 1a (2) indicates occurring casting defects (pores). The dark appearing primary Mg_2_Si phase in the LFe-LMn alloy (Figure 1a (3)) is also found in the other alloys. Particle feature analysis of both samples shows no indications for α-Al_15_(Fe,Mn)_3_Si_2_ phases nor denotes the Al_6_(Fe,Mn) phase. Details on area fraction and shape as well as the types of occurring phases are summarized in Table 2.

Figure 1b,d show the resulting primary phase distribution for LFe-HMn and HFe-HMn. With an increase in Mn contents (while keeping the Fe at 0.1 wt.%) the intermetallic particles show a significant change in shape and size (comparing Figure 1a and b). The above-mentioned needle-shaped, bright phase coarsens and tends to form structures of more complex geometry (Figure 1b (4)). EDX feature analysis also shows weak Si concentration (about 3 wt.%) in some of the primary phases of the LFe-HMn alloy. While the Si level is still too low to indicate a formation of the stoichiometric Al_15_(Fe,Mn)_3_Si_2_ phase, it implies the formation of some different AlFeSi-phase type.

The behavior of shape alteration is even more distinct in the HFe-HMn sample, where the primary intermetallics form networks of coarse phases. The platelet-like or Chinese script structures are uniformly found throughout the sample (Figure 1d (7)). Chemical analysis of the bigger primary phases using EDX indicates exclusively the Al_6_(Fe,Mn) phase forms because of the enhanced Mn content HFe-HMn.

The analysis of the dark phase shows Mg_2_Si particles for both LFe-HMn and HFe-HMn. Besides the majority of particles, some small high Fe containing particles with composition closer to Al_3_(Fe,Mn) were found in both alloys. As apparent in Figure 1b (5), the shape and size of those precipitates is different to the samples LFe-LMn and HFe-LMn.

The data given in Table 2, including over 1000 particles per sample, confirm the visual trend of primary phase formation in the samples. While the amount of dark appearing Mg_2_Si phases is not much different in all four samples, the total phase fraction is influenced only by the Fe- and Mn-containing phases. Surprisingly, both samples with either increased Fe or Mn contents show approximately equal fractions of Al-Fe or Al-Mn particles. Only the combined increase of Fe and Mn in sample HFe-HMn results in stronger primary precipitation and, therefore, a higher area fraction.

The Fe/Mn-ratio of the intermetallics is in good agreement with the samples alloying contents. Moreover, the higher Fe bearing phases show a clear tendency to form elongated phases, which is indicated by SF to a certain degree. However, since the Al_6_(Fe,Mn) particles in higher Mn containing alloys are also irregularly shaped, the shape factors can only be considered in combination with the micrographs.

#### 3.1.2. Near Rapid Cooling during Solidification (NR-C Cast)

**Figure 2:**
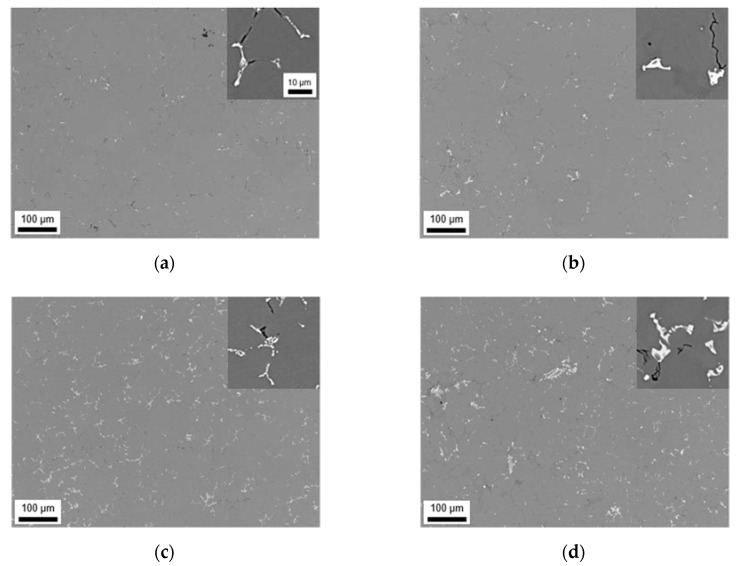
Casting microstructure of NR-C cast samples; (**a**) LFe-LMn, (**b**) LFe-HMn, (**c**) HFe-LMn, (**d**) HFe-HMn; the insert scale bar in (**b**–**d**) is the same as for (**a**).

The results of the NR-C cast samples are shown in Figure 2. The faster cooling clearly results in a smaller casting cell size and smaller primary phase particles by a factor of approximately 5 to 10. Beside this refinement there are lower area fractions of Mg_2_Si phase observable (compared to the S-C samples), which is also stated in Table 3. For an easier comparison see Table A1, which contains the fundamental data on the primary phases of various sample states.

The general trend of phase formation in LFe-LMn alloy is similar to the results for the S-C conditions during casting. The microstructure shows small needle-shaped phases with a higher Fe/Mn ratio (Table 3) and a netlike structure. Since the total area fraction of primary precipitates for this NR-C sample do not very much differ from the S-C numbers given in Table 2, the increase in Fe/Mn ratio of the particles implicates a higher Mn concentration in the aluminum matrix. The EDX data denote again the Al_3_(Fe,Mn) phase in the NR-C cast conditions.

For the LFe-HMn alloy in Figure 2b, a slight alteration of the microstructure is found with increased cooling rate. The primary phases tend to form more compact and spherical blocks without the dense netlike structure. Some particles seem disconnected in the 2D micrographs (Chinese-script structures), which also affects the SF data given in Table 3. Closer analysis of the phases reveals the Al_6_(Fe,Mn) phase in addition to Al_15_(Fe,Mn)_3_Si_2_ type, but the EDX analysis does not reflect the exact stoichiometry.

In HFe-LMn (Figure 2c), more Chinese-script phases instead of needles are found within the NR-C samples, whereas the chemical composition still points out Al_3_(Fe,Mn) phase type. The HFe-HMn alloy in Figure 2d also shows, besides the mentionable refinement, a trend towards spheroidization of the features. The phases were identified to be most likely Al_6_(Fe,Mn) with no remaining Al_3_(Fe,Mn) phase. Interestingly, similar AlFeSi phases are found in HFe-HMn alloy and LFe-HMn.

The area fractions and Fe/Mn ratios of the samples show further interesting results. For both high Mn containing samples, the total area fractions are lower for the NR-C conditions, whereas the number of primary phases increases in the HFe-LMn alloy. Since the Fe/Mn ratio of all NR-C samples is higher compared to the S-C samples (Table 2), a clear trend of inhibited Mn precipitation or increased Mn in solid solution can be assumed.

### 3.2. Microstructural Evolution during Homogenization

This section describes the microstructural changes upon two different homogenization treatments (500 and 550 °C). Each subsection includes the description of both the primary and secondary phase evolution as well as phase fractions and micrographs.

#### 3.2.1. Microstructure after Homogenization at 500 °C of S-C and NR-C Cast Samples

**Figure 3:**
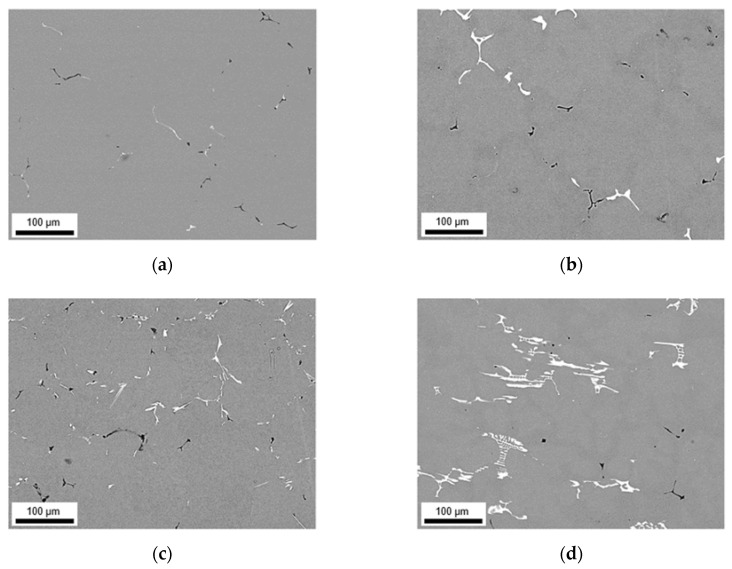
Microstructure and primary phase distribution of S-C cast samples after homogenization at 500 °C; (**a**) LFe-LMn, (**b**) LFe-HMn, (**c**) HFe-LMn, (**d**) HFe-HMn.

The homogenization leads to a slight decrease in total area fraction of the primary phases. In Figure 3, coarsened Al-Fe or Al-Mn phases are observed compared to as-cast conditions. Especially LFe-LMn and HFe-HMn samples show distinct coarsening of the before fine Al_3_(Fe,Mn) needles. For both high-Mn alloys (Figure 3b,d), the smaller precipitates show more spherical shaped structures, but especially the HFe-HMn alloy shows some increase of the Chinese-script structures.

The visible trends of phase alteration are confirmed by the data given in Table 4. In comparison to the as-cast samples, the total primary phase fraction and simultaneously both the Fe/Mn-bearing as well as Mg_2_Si phases are decreased. Concerning the Fe/Mn-ratio, the high Mn containing alloys show no significant changes, although the standard deviation becomes significantly smaller. The two high Fe containing alloys show some slight increase in the Fe/Mn-ratio. Detailed consideration reveals the increase of average Fe contents in the primary phases but no significant change in Mn concentration. The EDX feature mapping indicates no transformation of primary phases. The changes in shape are also indicated by the slight decrease of SF comparing the results to the S-C cast samples in Table 2, Table 3 and Table 4.

**Figure 4:**
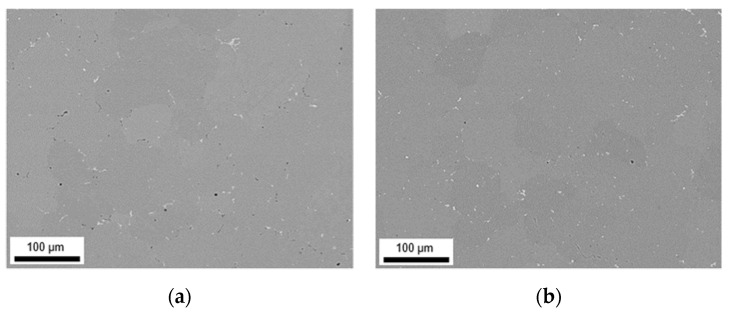

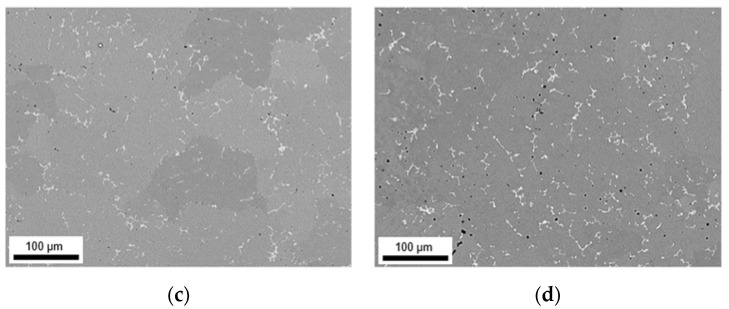
Microstructure and primary phase distribution of NR-C cast samples after homogenization at 500 °C; (**a**) LFe-LMn, (**b**) LFe-HMn, (**c**) HFe-LMn, (**d**) HFe-HMn.

Figure 4 shows the micrographs for the NR-C cast samples homogenized at 500 °C. The microstructural characterization leads to results similar as for the S-C cast samples. However, the HFe-HMn sample shows coarsening and an increase in total primary phase fraction (Figure 4d). Additionally, the small number of Chinese-script phases found in the as-cast state is further reduced, since there are no such features observable anymore. The micrographs in Figure 4 (mainly HFe-HMn in Figure 4d) show small, circular black phases which were identified as pores using EDX (matrix signal).

Comparing primary phase data of Table 2 and Table 4, tendencies of reduction in total phase fraction can be observed for all samples except HFe-HMn. Interestingly, the Fe/Mn ratio of the precipitates is modified to lower values, which contrasts with S-C cast conditions. The EDX data denote slight concentrations of Mn in connection with reductions in Fe contents, but no phase transformations in general. Finally, the primary phases do not show the clear trend of spheroidization as noticeable before for the S-C cast and homogenized samples.

**Figure 5:**
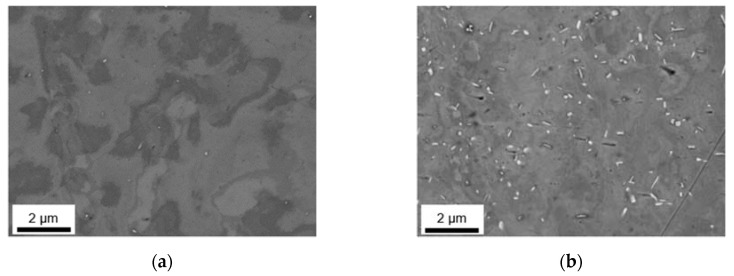

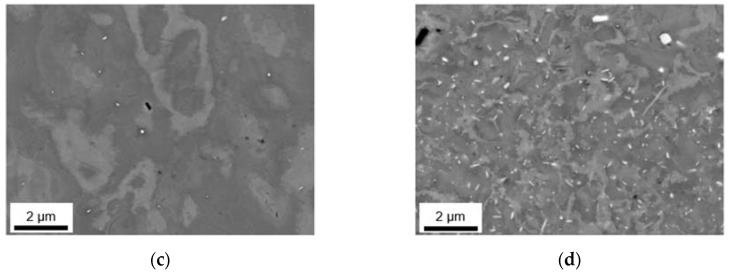
Secondary phase distribution of S-C cast samples after homogenization at 500 °C; (**a**) LFe-LMn, (**b**) LFe-HMn, (**c**) HFe-LMn, (**d**) HFe-HMn.

**Figure 6:**
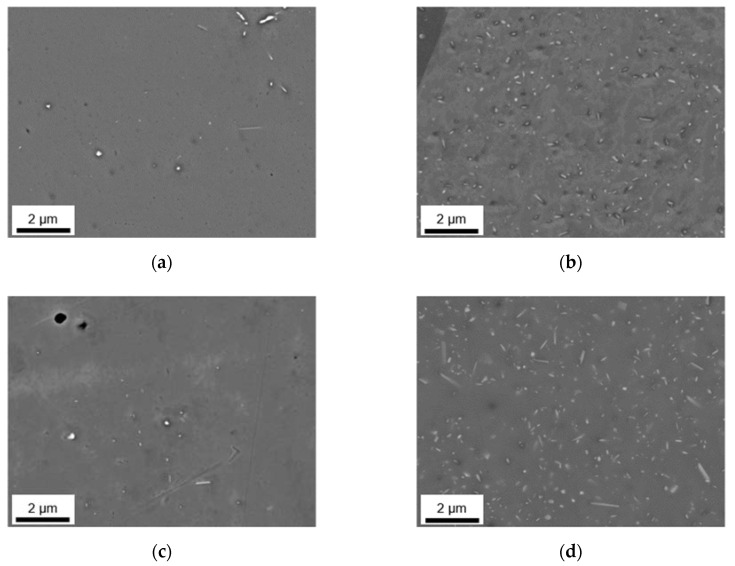
Secondary phase distribution of NR-C cast samples after homogenization at 500 °C; (**a**) LFe-LMn, (**b**) LFe-HMn, (**c**) HFe-LMn, (**d**) HFe-HMn.

Besides the alteration of the primary phase number density and size, the homogenization initiates the formation of secondary phases. The analysis of dispersoids followed the methods described in Section 2 using ImageJ software. Figure 5 and Figure 6 show the microstructural features after homogenization at high magnification for S-C cast and NR-C cast samples, respectively. In both figures, clear trends of dispersoid formation in dependence on the alloying contents are observable. The obtained blurred contrast variations in the background in Figure 5 are attributed to the preparation and not to microstructural conditions in the sample. However, the emergence of the contrast is not yet clarified.

For both low Mn alloys, the number density of secondary particles (given in Table 5) is very low. While there is no difference in secondary phase precipitation perceivable with the change in Fe contents for LFe-LMn and HFe-LMn for the homogenized S-C cast samples, the total area fraction of dispersoids for the HFe-LMn alloy is approximately two times higher in the NR-C cast LFe-LMn alloy after homogenization. The micrographs in Figure 5 and Figure 6a,c illustrate this trend. Furthermore, they show a slight modification of dispersoids shape from spheroidal to rod-shaped with the different casting conditions. This change is tabulated in the mean aspect ratio (AR) of the particles in Table 5, where an increase of AR is especially noticeable for LFe-LMn.

In both alloys with high Mn content, the dispersoid formation is clearly visible (Figure 5b,d). Comparing the NR-C and S-C casting conditions (see Figure 5 and Figure 6), the only clearly observable difference is a slight refinement of the secondary phase particles for the LFe-HMn alloy. This accords with the similar total area fraction of the differently casted samples, while r decreases significantly (Table 5). The AR of the samples show no change and confirm the rod-shape of the particles.

The results for HFe-HMn are in good agreement with the statements for LFe-HMn above. The micrographs (Figure 5d and Figure 6d) depict high similarities of the dispersoids shape in dependence on the prior casting procedure, whereas the volume fraction is clearly higher for NR-C casting conditions, which is also verified by the data of the total volume fractions of dispersoids in Table 5. While there is no difference in the secondary phase morphology deducible from the AR data given, a small increase in the radius of the dispersoids is obtained for the NR-C conditions.

#### 3.2.2. Microstructure after Homogenization at 550 °C of S-C and NR-C Cast Samples

**Figure 7:**
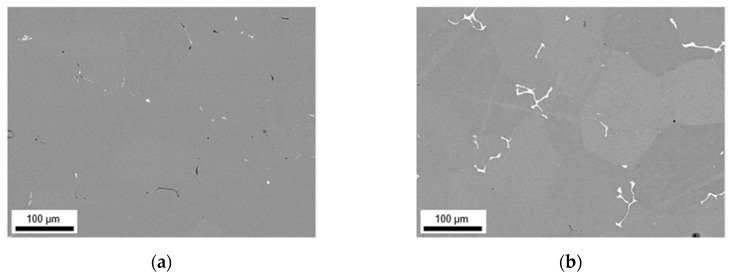

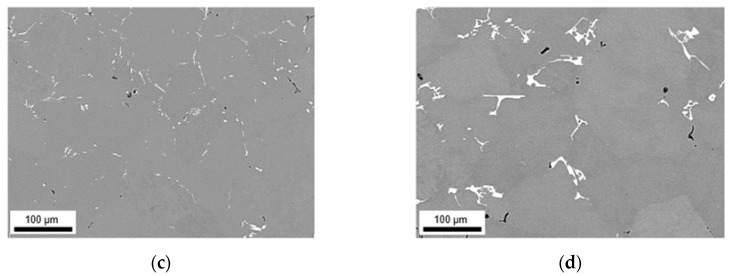
Microstructure and primary phase distribution in S-C cast samples after homogenization at 550 °C; (**a**) LFe-LMn, (**b**) LFe-HMn, (**c**) HFe-LMn, (**d**) HFe-HMn.

Most of the typical primary phase particles found in Figure 1 persist the high temperature homogenization. However, a large extent of primary Mg_2_Si is dissolved, evident in Figure 7a–d by less dark appearing phases and Table 6 showing a large reduction in the dark area fractions due to homogenization for samples cast under S-C conditions.

In the LFe-LMn alloy (Figure 7a), the Al_3_(Fe,Mn) precipitates do not show distinct alteration. The micrograph indicates the tendency of the primary phases to become finer and spheroidized with the homogenization. The data given in Table 6 show, besides a reduction in overall primary phase fraction, a slight decrease in Fe/Mn ratio, whereas the shape factor of the particles remain nearly unchanged. This change in Fe/Mn ratio and the atomic ratios point towards a beginning phase transformation from Al_3_(Fe,Mn) to Al_6_(Fe,Mn).

In the LFe-HMn alloy (Figure 7), the typical Al_15_(Fe,Mn)_3_Si_2_ primary phases are conserved. In addition to the original primary phases, some medium sized (few microns) secondary phases precipitate during homogenization. With its slightly raised Si concentration, these phases exhibit correlations to α-Al_15_(Fe,Mn)_3_Si_2_. The data displayed in Table 6 show, apart from the considerable decrease in Fe/Mn ratio, a modification of their shape in contrast to the as-cast state.

While the HFe-LMn alloy in Figure 7c shows some equivocal refinement of the needle-like Al_3_F(Fe,Mn) precipitates, the network structure of phases in HFe-HMn becomes looser and particles less branched. As stated in Table 6, the phases merely change in shape and number density. In neither of both high Fe alloys are phase transformations traceable.

**Figure 8:**
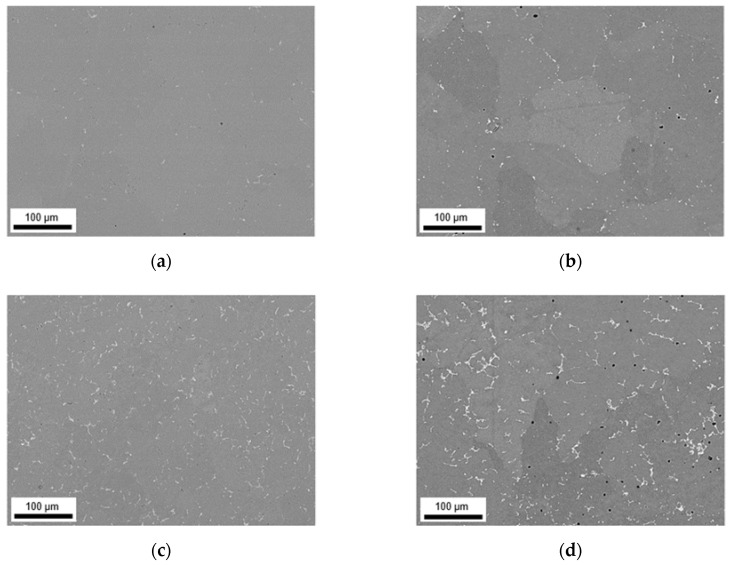
Microstructure and primary phase distribution in NR-C cast samples after homogenization at 550 °C; (**a**) LFe-LMn, (**b**) LFe-HMn, (**c**) HFe-LMn, (**d**) HFe-HMn.

For the NR-C cast and homogenized alloys (Figure 8a–d), the trend of Mg_2_Si dissolution is retained. The types of phases obtained conform with the initial situation in the samples, at which no transformations are perceived.

The LFe-LMn alloy does not show significant differences induced by the higher temperature homogenization, noticeable when comparing Figure 8a to Figure 2a or Figure 4a. According to Table 6, the Fe/Mn ratio decreases significantly, and the shape factor indicates weak spheroidization. The reduction of the total primary phase fraction can be attributed to Mg_2_Si dissolution.

For the LFe-HMn alloy, refinement of the former plate-like structures can be observed. According to the change in Fe/Mn ratio (compare Table 2 and Table 6), an integration of solute Mn from the matrix into the phases is to be assumed, as the average Mn level in the primary phases rises.

For both high-Fe variants, a looser structured network of intermetallic phases can be noticed. A reduction in Fe/Mn ratios is perceivable for both alloys. While the total fraction of primary phases is reduced or at least kept in the same range for HFe-LMn, the alloy with high Fe and high Mn contents exhibits an increase of the total area fraction. The reduction of the shape factor values for both alloys portends spheroidization of the particles (Table 6).

**Figure 9:**
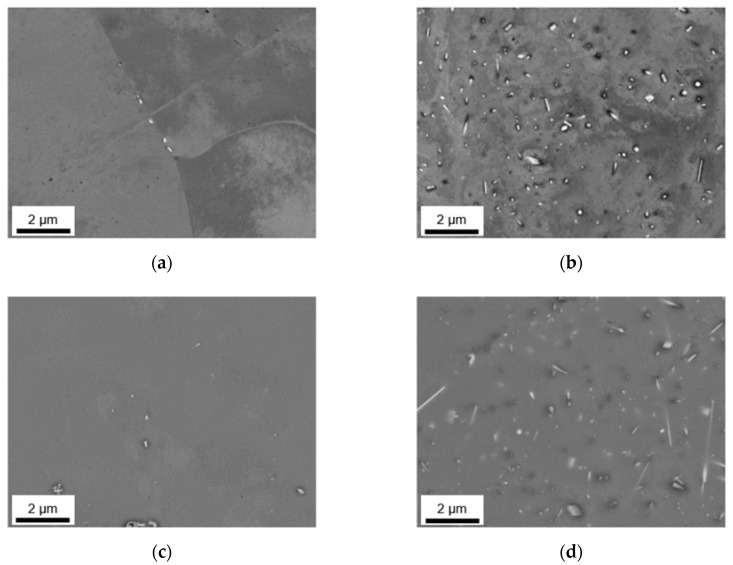
Secondary phase distribution in S-C cast samples after homogenization at 550 °C; (**a**) LFe-LMn, (**b**) LFe-HMn, (**c**) HFe-LMn, (**d**) HFe-HMn.

**Figure 10:**
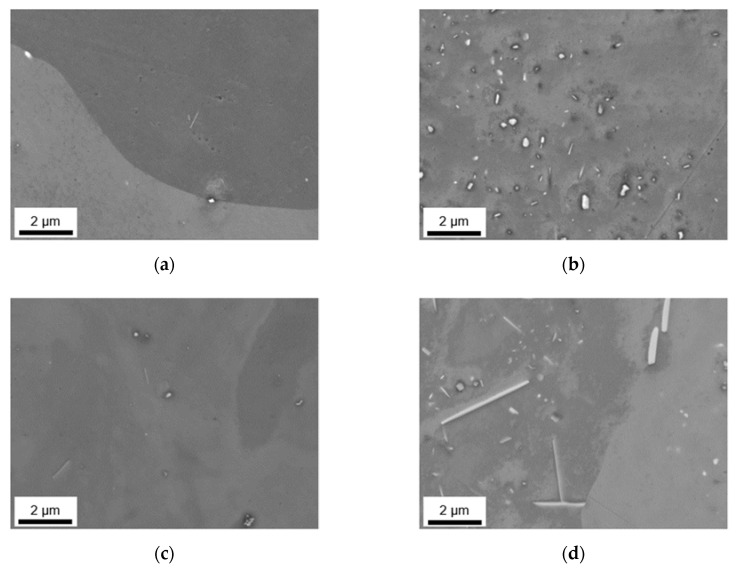
Secondary phase distribution of NR-C cast samples after homogenization at 550 °C; (**a**) LFe-LMn, (**b**) LFe-HMn, (**c**) HFe-LMn, (**d**) HFe-HMn.

Figure 9 and Figure 10 show the secondary phase evolution as a result of the 550 °C homogenization in high resolution for S-C and NR-C cast material, respectively. The precipitation of the dispersoids shows similar results concerning the dependence on Fe and Mn alloying contents as after the 500 °C homogenization. From the micrographs in Figure 9 and Figure 10, coarsening of the secondary phases is perceptible at higher temperatures, while the block- or rod-shape for high Mn containing alloys is preserved.

Data on secondary phase number density as well as size and shape are given in Table 7 (or in Table A2 for easier comparison to the 500 °C homogenization). The total area fraction increases for the HFe-LMn alloy cast under NR-C conditions, while the area fractions of the other alloys do not show variations with the cooling rate.

Coarsening of the dispersoids after homogenization at 550 °C is affirmed by the average radii (note the values in Table 5). Furthermore, the mix of high Fe and high Mn contents in combination with higher cooling rates results in the formation of micron sized, rod-shaped secondary phases, which is quantified by the reduced aspect ratio (Figure 10d).

#### 3.2.3. Phase Characterization Using TEM

**Figure 11:**
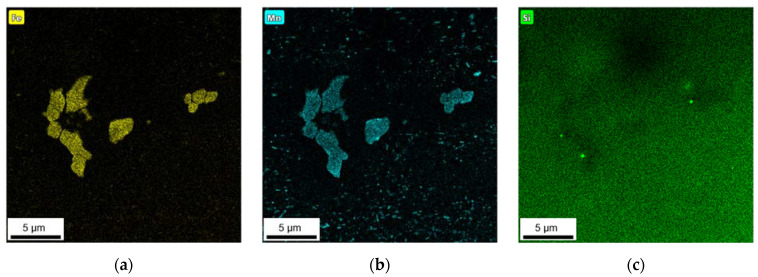

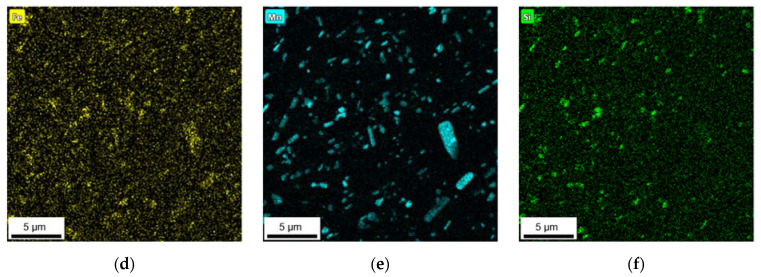
EDX maps of the HFe-HMn alloys with NR-C (**a**–**c**) or S-C (**d**–**f**) casting conditions after homogenization at 500 °C from TEM measurements; (**a**,**d**): Fe map, (**b**,**e**) Mn map, (**c**,**f**) Si map.

To verify the composition and type of the obtained primary and secondary phases with the SEM-EDX feature mappings and point analyses, TEM measurements were performed on the HFe-HMn alloy. The resulting TEM EDX maps for the Fe, Mn and Si signal are shown in Figure 11.

The micron sized primary phase particles show enhanced intensities of Fe and Mn, while there is no evidence of Si. The Fe distributions shown in Figure 11a,d further indicate the integration of iron in secondary phase particles. However, as shown in Figure 11b,e, the elemental allocation of manganese reveals a high number of dispersoids without Fe. Meanwhile, Figure 11c,f depict the general distribution of silicon in the sample’s microstructural features. No indications for Si enrichment are visible in any of the primary or secondary phases in the NR-C cast HFe-HMn alloy (Figure 11c). However, fine Si containing dispersoids are present in the homogenized S-C samples (Figure 11f).

### 3.3. Thermodynamic Calculations

In addition to the experimental data given in Section 3.1 and Section 3.2, this section provides information on the phase formation computed using the thermodynamic database PanAl2019. The simulation was performed for the four different alloys used in this study and the results are shown in Table 8. Each column and row give the fractions of elements (in wt.%) or phases (in vol.%) present after casting (approximated by a nonequilibrium Scheil calculation) and 550 °C homogenization (approximated by equilibrium calculation).

The first two columns of Table 8 contain the information of solute elements Fe and Mn in the fcc matrix. Generally, the significantly higher solute content of manganese is evident in all alloys. The fractions of the dissolved elements change with the general alloying contents. Furthermore, there is an interdependent influence of Fe and Mn observed, since higher solution of one of the elements reduces the solubility of the other one. The comparison of nonequilibrium and equilibrium calculations shows the considerable reduction of solute contents, especially for the higher Mn alloyed samples.

Moreover, Table 8 contains the information of primary and secondary phase evolution in terms of the thermodynamic aspects. The LFe-LMn alloy in as-cast state only shows occurrence of Al_3_(Fe,Mn) (considered as equivalent to Al_13_(Fe,Mn)_4_ in Pandat). With the homogenization the primary phases are either dissolved or transformed, since in the equilibrium state only Al_6_(Fe,Mn) is predicted to remain. The volume fraction data also include the fine dispersed secondary phase particles, which in turn shows a very low precipitation of phases during homogenization in this alloy. The transformation of primary phases or the precipitation of dispersoids is also concordant with the decrease in solute contents of Fe and Mn in the LFe-LMn alloy. Similar results are obtained for the HFe-LMn alloy. Primary phases before homogenization only consist of type Al_3_(Fe,Mn), which are transformed to some extent into Si containing Al_15_(Fe,Mn)_3_Si_2_ but not into Al_6_(Fe,Mn). The total volume fraction of phases increases from nonequilibrium to equilibrium conditions on the expense of solute elements due to the formation of dispersoids either of type Al_3_(Fe,Mn) or Al_15_(Fe,Mn)_3_Si_2_.

Considering the higher Mn containing alloys, different types of primary phases can occur, although the majority consists of Al_6_(Fe,Mn) for the low Fe variant. For higher Fe additions the volume fractions are almost evenly distributed among the different types of phases. With homogenization of the LFe-HMn sample, all Al_3_(Fe,Mn) particles are dissolved or transformed into other types. The strong precipitation of Al_15_(Fe,Mn)_3_Si_2_ and Al_6_(Fe,Mn) as dispersoids takes place mainly at the expense of the solute Fe and Mn. The HFe-HMn alloy shows, besides the distinct transformation or degradation of Al_3_(Fe,Mn), a strong tendency of secondary Al_15_(Fe,Mn)_3_Si_2_ phase precipitation, even though Al_6_(Fe,Mn) also represents a significant phase fraction.

### 3.4. Soft Annealed State

**Figure 12:**
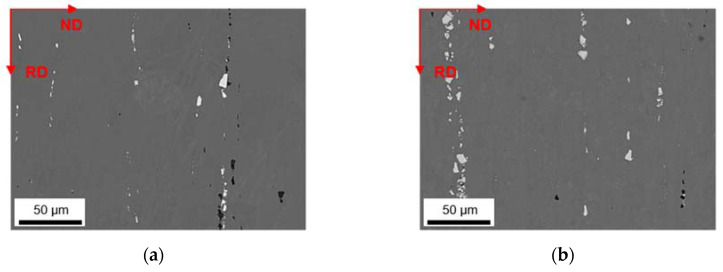

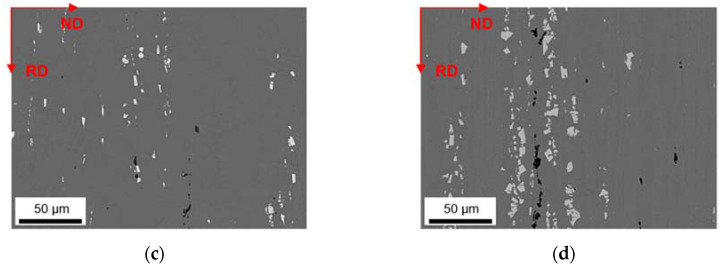
Soft annealed microstructure of the alloys cast under S-C conditions, homogenized at 500 °C and cold rolled to a CRD of 63%; (**a**) LFe-LMn, (**b**) LFe-HMn, (**c**) HFe-LMn, (**d**) HFe-HMn; RD: rolling direction, ND: normal direction.

**Figure 13:**
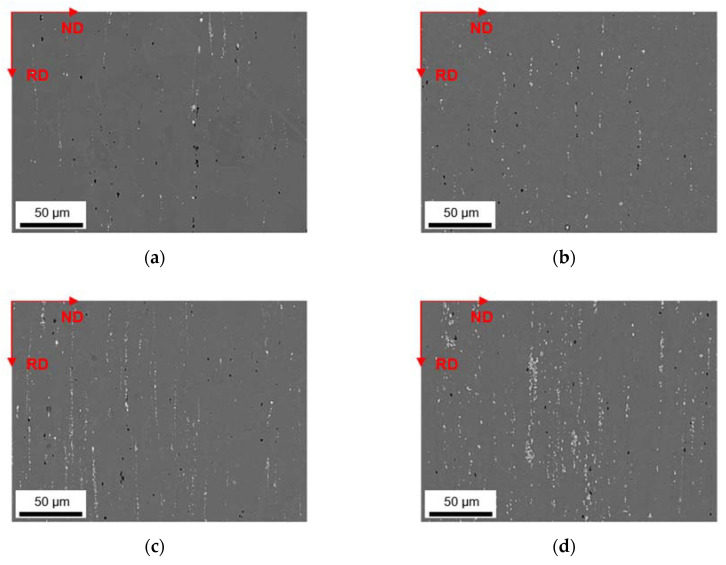
Soft annealed microstructure of the alloys cast under NR-C conditions, homogenized at 500 °C and cold rolled to a CRD of 63%; (**a**) LFe-LMn, (**b**) LFe-HMn, (**c**) HFe-LMn, (**d**) HFe-HMn.

**Figure A6:**
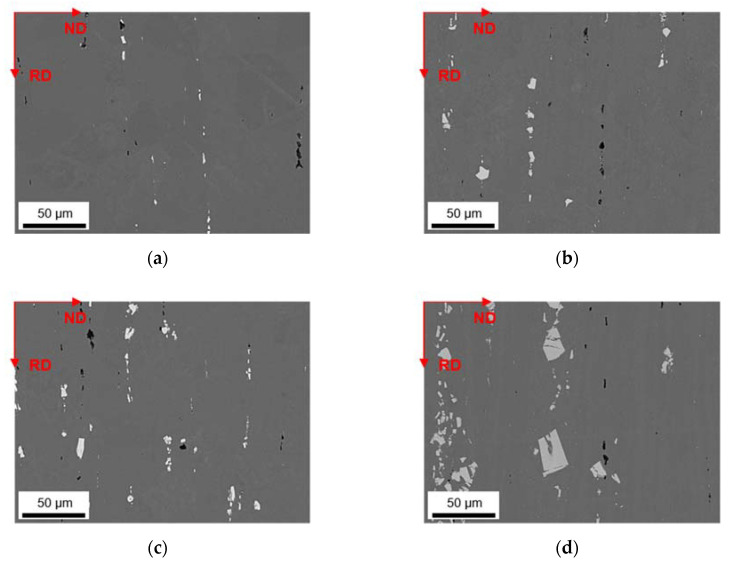
Soft annealed microstructure of the alloys cast under S-C conditions, homogenized at 500 °C and cold rolled to a CRD of 35%; (**a**) LFe-LMn, (**b**) LFe-HMn, (**c**) HFe-LMn, (**d**) HFe-HMn.

**Figure A7:**
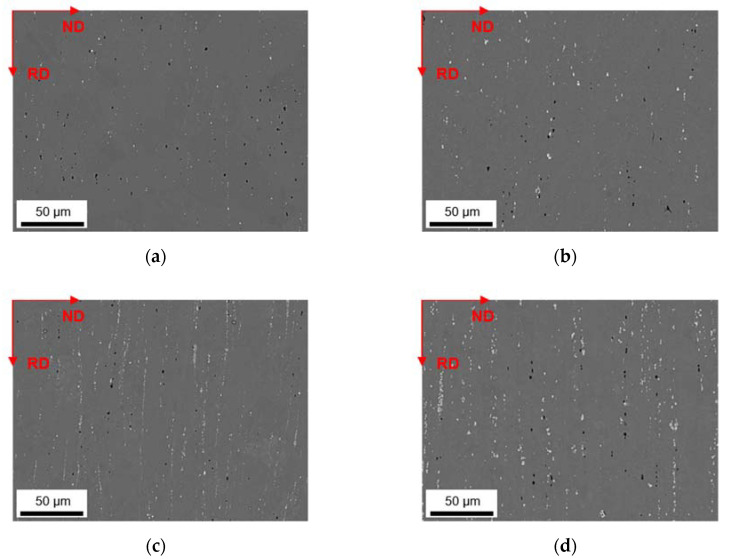
Soft annealed microstructure of the alloys cast under NR-C conditions, homogenized at 500 °C and cold rolled to a CRD of 35%; (**a**) LFe-LMn, (**b**) LFe-HMn, (**c**) HFe-LMn, (**d**) HFe-HMn.

**Figure A8:**
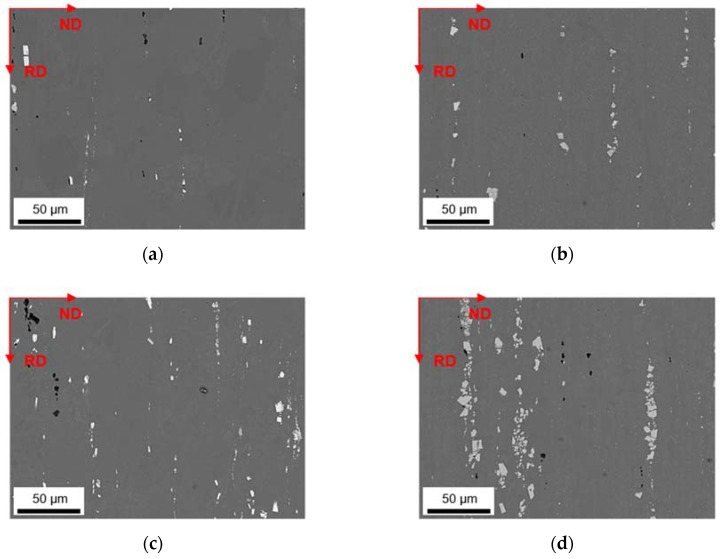
Soft annealed microstructure of the alloys cast under S-C conditions, homogenized at 550 °C and cold rolled to a CRD of 63%; (**a**) LFe-LMn, (**b**) LFe-HMn, (**c**) HFe-LMn, (**d**) HFe-HMn.

**Figure A9:**
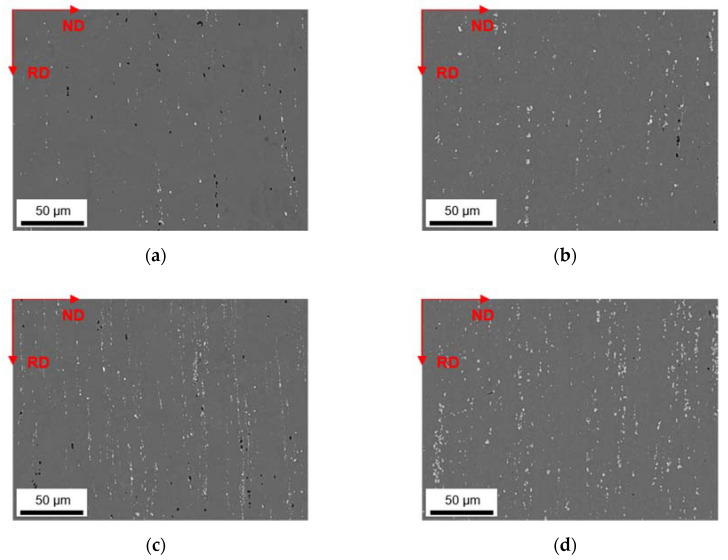
Soft annealed microstructure of the alloys cast under NR-C conditions, homogenized at 550 °C and cold rolled to a CRD of 63%; (**a**) LFe-LMn, (**b**) LFe-HMn, (**c**) HFe-LMn, (**d**) HFe-HMn.

**Figure A10:**
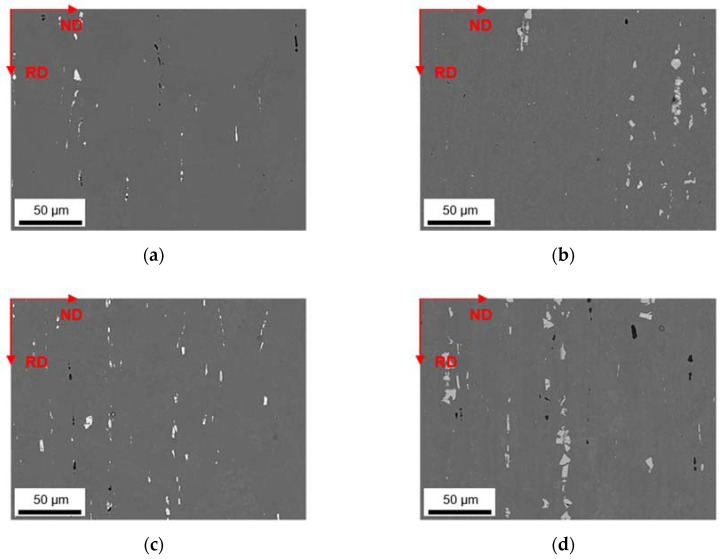
Soft annealed microstructure of the alloys cast under S-C conditions, homogenized at 550 °C and cold rolled to a CRD of 35%; (**a**) LFe-LMn, (**b**) LFe-HMn, (**c**) HFe-LMn, (**d**) HFe-HMn.

**Figure A11:**
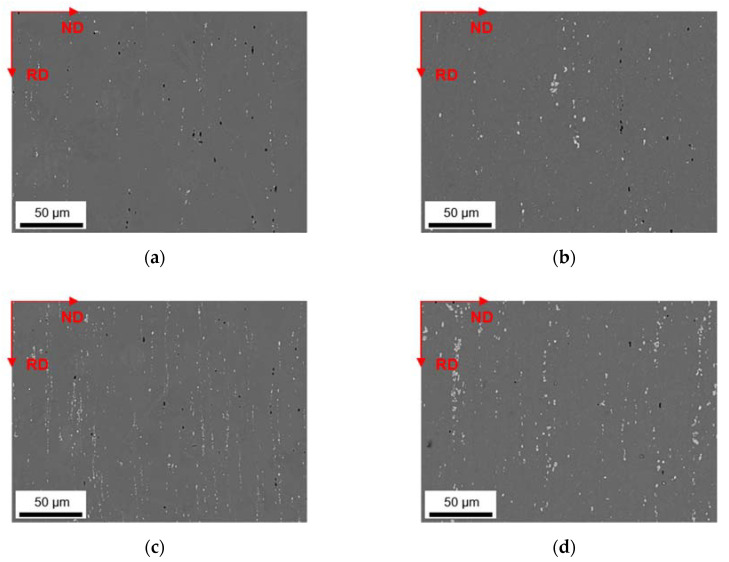
Soft annealed microstructure of the alloys cast under NR-C conditions, homogenized at 550 °C and cold rolled to a CRD of 35%; (**a**) LFe-LMn, (**b**) LFe-HMn, (**c**) HFe-LMn, (**d**) HFe-HMn.

The primary phases of the different alloys in the final soft annealed state (500 °C/5 min/H_2_O) are compared for S-C (Figure 12) and NR-C (Figure 13) conditions, homogenized at 500 °C, and cold rolled to a CRD of 63%. Further micrographs for the various homogenization temperatures and cold rolling degrees are shown in the Appendix A from Figure A6, Figure A7, Figure A8, Figure A9, Figure A10 and Figure A11. The primary phases are now fragmented and arranged in bands. Following the results of analyzed casting and homogenization samples, the area fraction of the primary phases depicts equal trends of increased number density with high Mn contents (comparing Figure 12a and Figure 13a to Figure 12b and Figure 13b or Figure 12c and Figure 13c to Figure 12d and Figure 13d) and increased sizes with high Fe contents (comparing Figure 12a and Figure 13a to Figure 12c and Figure 13c or Figure 12b and Figure 13b to Figure 12d and Figure 13d), and does not indicate significant changes during further rolling and soft annealing.

During rolling the rather needle- or rod-shaped particles in particular tend to align in the rolling direction. The initially different primary phase sizes (that result from the variations in the casting cooling rates) are retained even after final processing.

**Figure 14:**
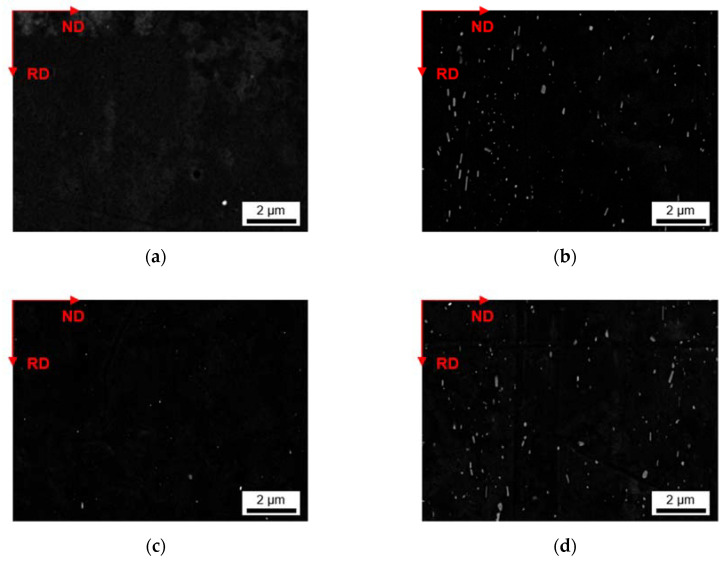
Dispersoids in the soft annealed sample state cast under S-C conditions, homogenized at 500 °C and cold rolled to a CRD of 63%; (**a**) LFe-LMn, (**b**) LFe-HMn, (**c**) HFe-LMn, (**d**) HFe-HMn.

**Figure 15:**
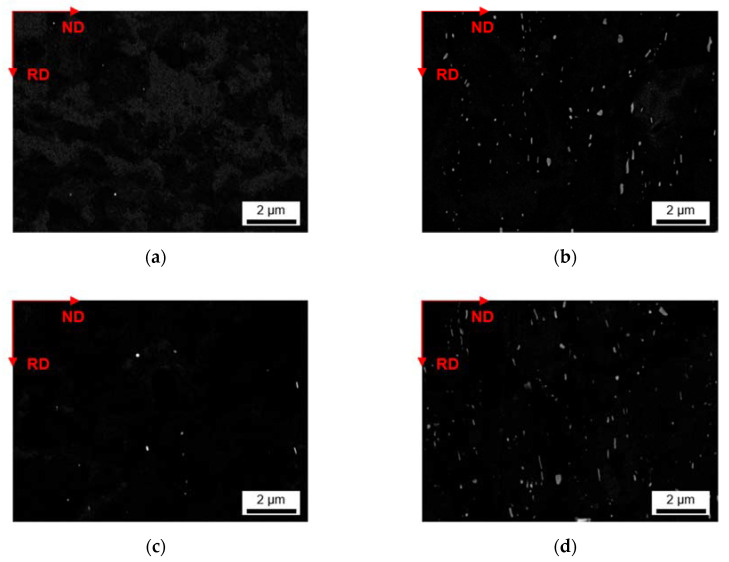
Dispersoids in the soft annealed sample state cast under NR-C conditions, homogenized at 500 °C and cold rolled to a CRD of 63%; (**a**) LFe-LMn, (**b**) LFe-HMn, (**c**) HFe-LMn, (**d**) HFe-HMn.

**Figure A12:**
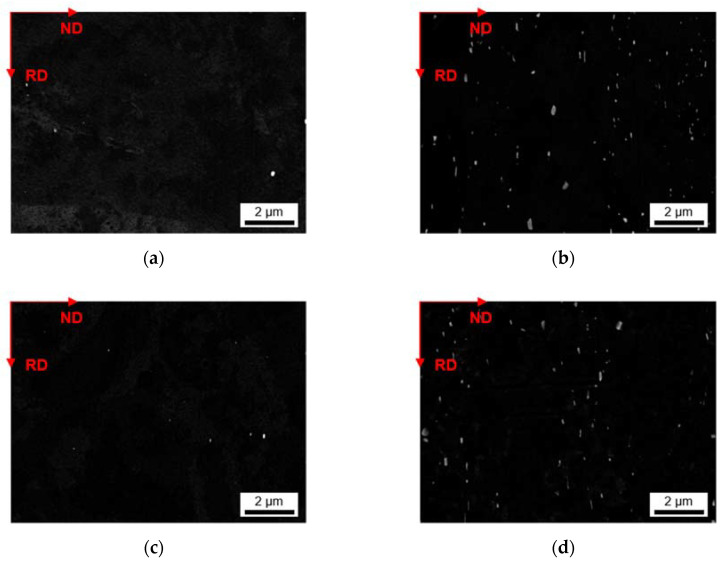
Dispersoids in the soft annealed sample state cast under S-C conditions, homogenized at 500 °C and cold rolled to a CRD of 35%; (**a**) LFe-LMn, (**b**) LFe-HMn, (**c**) HFe-LMn, (**d**) HFe-HMn.

**Figure A13:**
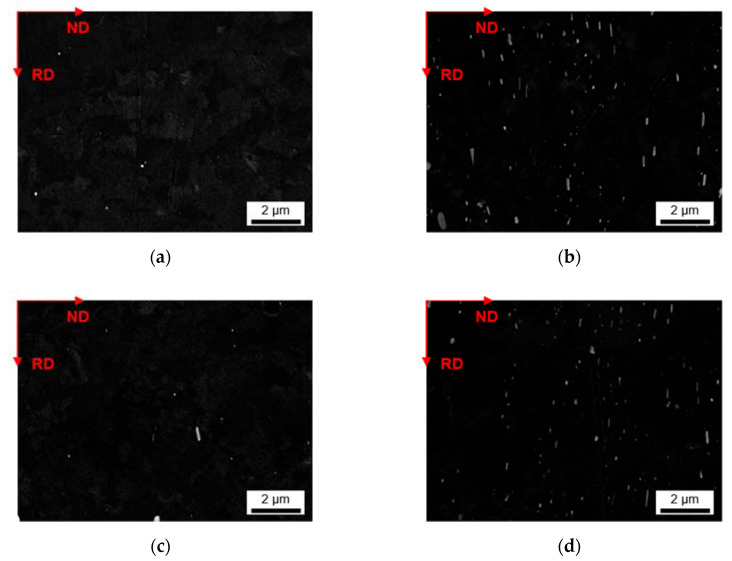
Dispersoids in the soft annealed sample state cast under NR-C conditions, homogenized at 500 °C and cold rolled to a CRD of 35%; (**a**) LFe-LMn, (**b**) LFe-HMn, (**c**) HFe-LMn, (**d**) HFe-HMn.

**Figure A14:**
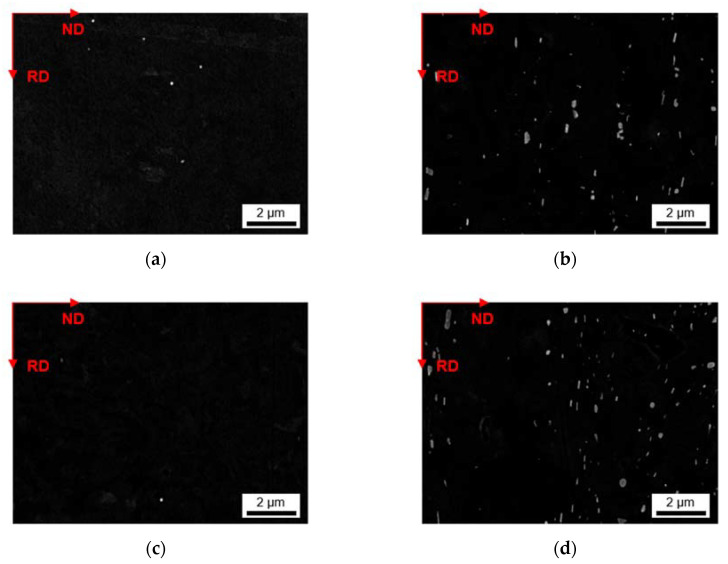
Dispersoids in the soft annealed sample state cast under S-C conditions, homogenized at 550 °C and cold rolled to a CRD of 63%; (**a**) LFe-LMn, (**b**) LFe-HMn, (**c**) HFe-LMn, (**d**) HFe-HMn.

**Figure A15:**
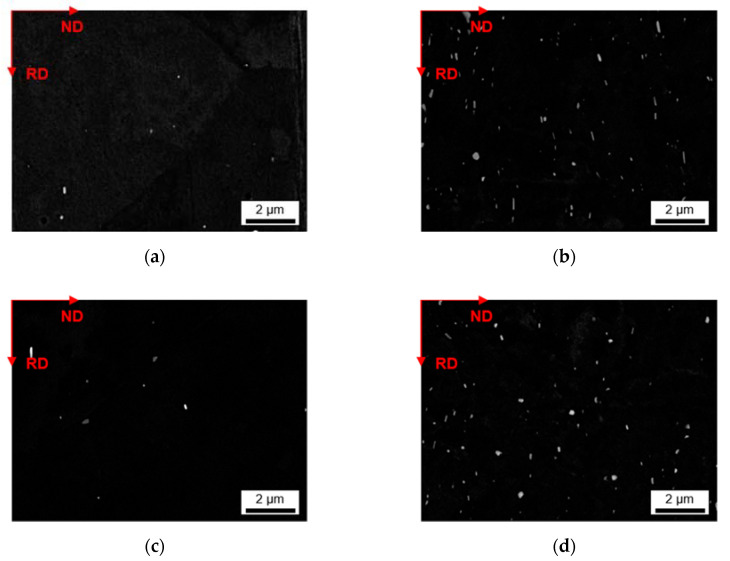
Dispersoids in the soft annealed sample state cast under NR-C conditions, homogenized at 550 °C and cold rolled to a CRD of 63%; (**a**) LFe-LMn, (**b**) LFe-HMn, (**c**) HFe-LMn, (**d**) HFe-HMn.

**Figure A16:**
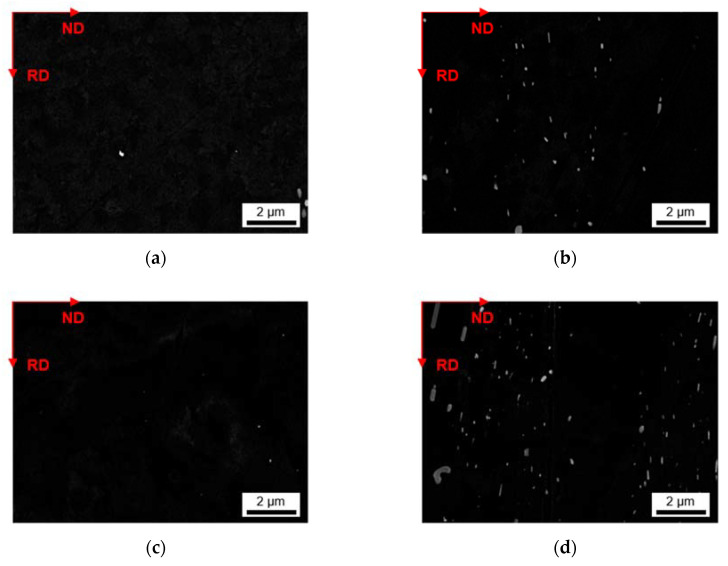
Dispersoids in the soft annealed sample state cast under S-C conditions, homogenized at 550 °C and cold rolled to a CRD of 35%; (**a**) LFe-LMn, (**b**) LFe-HMn, (**c**) HFe-LMn, (**d**) HFe-HMn.

**Figure A17:**
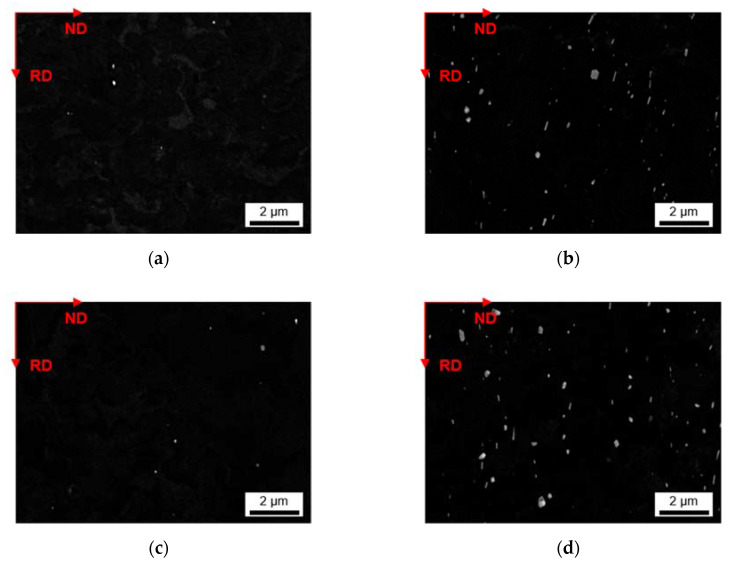
Dispersoids in the soft annealed sample state cast under NR-C conditions, homogenized at 550 °C and cold rolled to a CRD of 35%; (**a**) LFe-LMn, (**b**) LFe-HMn, (**c**) HFe-LMn, (**d**) HFe-HMn.

The alignment of the dispersoids in rolling direction is shown for exemplary sample states in Figure 14 and Figure 15. The micrographs of the additional 24 sample states can be found in the Appendix A (Figure A12, Figure A13, Figure A14, Figure A15, Figure A16 and Figure A17). The trends of favored dispersoid formation with increased Mn contents (as observed after homogenization) are still clearly visible comparing Figure 14a or Figure 15a to Figure 14b or Figure 15b. However, the figures indicate a slightly higher dispersoid number density with S-C cooling conditions in the soft annealed sample state.

Additional information is given by the volume fractions and morphological parameters of the dispersoids in Table 9 (S-C cast conditions; all sample states) and Table 10 (NR-C cast conditions; all sample states). The higher average volume fraction for the S-C cast samples is verified. Comparing the data to the homogenized sample states (Table 5 and Table 7), a clear trend towards lower volume fractions after further processing is obtained.

While the increase of the average dispersoid radius is still observed for the higher homogenization temperature, the total dispersoid radii are clearly shifted to lower values. The rod-shaped particle morphology is however still maintained as expressed by the AR data given in Table 9 and Table 10.

## 4. Discussion

In general, the types and fractions of the occurring intermetallic phases reflect the composition of the alloys. If one first looks at the S-C cast samples in Figure 1, the primary phases of LFe-LMn and HFe-LMn only contain low concentrations of Mn. The characteristic needle-like shape and composition clearly indicate the Al_3_(Fe,Mn) phase, which is in good agreement with the results from other studies [6,9,10] and thermodynamic simulation. The stronger segregation of iron in comparison to Mn is based on the low solubility of Fe in the Al matrix [17,18,22]. Therefore, the increase in Fe alloying contents promotes higher number densities of primary phase nuclei, whereas the shape and structure of the phases remain unaltered.

With the increase in manganese alloying content, a significant change in shape of the primary phases can be observed. The needle-like intermetallic phases coarsen and form block-like structures in LFe-HMn or branched and Chinese-script structures in HFe-HMn, which was similarly observed by Refs. [9,12,19]. With the change in Fe/Mn alloying ratio and due to the interchangeability of Fe and Mn, the stable eutectic component (precipitating as primary phase particles) becomes Al_6_(Fe,Mn) instead of Al_3_(Fe,Mn) [18]. While in [12,16] these changes were similarly observed especially in consideration of the increased Mn contents, Li and Arnberg [9] contradict this as high Mg contents in the alloys largely prevent the formation of the Al_6_(Fe,Mn) phase. The authors state preferred formation of Al_3_(Fe,Mn) and Al_m_(Fe,Mn) with m ranging from 4.1 to 4.4, but also suggest higher stability for Al_6_(Fe,Mn) with increasing Mn content. Since the Mn level in the investigated alloys is significantly higher, the described effects of Mg on the Al_m_(Fe,Mn) phase formation will presumably be retarded.

In addition to the Al_6_(Fe, Mn) phases, particles with a higher Si concentration (classified as non-stoichiometric Al_15_(Fe,Mn)_3_Si_2_) were found for the LFe-HMn alloy under S-C cast conditions. The alteration of phase stability is attributed to the very low Fe/Mn ratio of the LFe-HMn alloy, which was also observed in other studies for very similar compositions [6,10].

Liu et al. [12] describe the effects of Mg and Si contents on the precipitation behavior of the Fe-Mn phases. The formation of (coarse) Mg_2_Si, which was also observed in the present work in all alloys, subsequently affects the precipitation behavior of the Fe-Mn phases. With the decrease in average Si concentration in the matrix due to precipitation of Mg_2_Si, the Si-containing Fe-Mn phases can hardly be formed. The absence of the non-stoichiometric Al_15_(Fe,Mn)_3_Si_2_ phase for HFe-HMn results from both the change in Fe/Mn ratio and the Mg_2_Si precipitation from the melt. A partial dissolution of Mg_2_Si was observed for various alloys in the further homogenization processes at different temperatures [10,21].

Numerous studies already mentioned the importance of the cooling rate in connection with the phases formed in a large variety of alloys [7,9,12,14,16]. Comparing Figure 1 and Figure 2, a significant reduction in the casting cell size as well as the primary phase size by a factor of 5 to 10 is observable for the NR-C conditions. Furthermore, especially for Al_3_(Fe,Mn) containing alloys, an alteration of the needle shape can be observed (Figure 2a). The fast solidification decreases the casting cell size and therefore the final solidification zones, which become smaller but more branched [12,16]. The near rapid cooling promotes therefore a higher number density of primary phases, whereas their total area fraction remains constant. As the mean area of the individual primary phases is reduced, the detectability of the lamellar structure is restricted. Furthermore, higher cooling rates favor the formation of Chinese-scripted phases also in low Mn containing alloys (Figure 2c).

In addition to the shape modifications of the intermetallic phases, the fractions of the primary phases for the different cooling conditions are compared in Table 2 and Table 3. Like in [7,12,16], the clear increment in the total phase fraction is a result of the higher alloying contents of Fe and/or Mn. A trend of increased primary phase fractions with lower cooling rates can be noticed for most of the alloys and was also found by [7]. The contrary trend for the HFe-LMn alloy is again connected to the low solubility of Fe in the matrix, indicated by the change in Fe/Mn ratio of the primary phases with varied cooling conditions. Higher values for the Fe/Mn ratio indicate higher solute concentrations of Mn in the surrounding Al matrix or an increased segregation of Fe. Besides the alteration in Fe/Mn ratio, NR-C also stabilizes the non-stoichiometric Al_15_(Fe,Mn)_3_Si_2_ phase in the HFe-HMn alloy. A possible explanation might be the higher level of solute Si retained during solidification since the fraction of Mg_2_Si is considerably lower in comparison to S-C conditions.

The homogenization heat treatment only induces a phase transformation for the S-C cast LFe-HMn at a temperature of 550 °C (Al_3_(Fe,Mn) to Al_6_(Fe,Mn)) [21,22]. The frequently examined phase transformation from disadvantageous Fe- and Mn-bearing phases to α-Al_15_(Fe,Mn)_3_Si_2_ is not observed in the present alloys, most probably because of the low Si content [22]. The effect of the homogenization treatments on the total fraction of the primary phases can be noticed when comparing Table 2, Table 3, Table 4, Table 5 and Table 6. When considering the Fe-Mn bearing phases, the homogenization at 500 °C noticeably reduces the fraction of those phases. The higher temperature of 550 °C increases the maximum solubility of Fe and Mn in the matrix and diffusion enables slight dissolution. However, with higher homogenization temperatures, an increase in the area fraction is obtained for all alloys. Since this behavior contrasts with expectations, the fractions for the primary phases in Table 4 and Table 6 may also include parts of the secondary phase fractions.

Furthermore, as reported in [10], the homogenization can cause both shrinkage or growth of the constituent phases as well as spheroidization [26,34], which is depicted in Figure 3, Figure 4, Figure 7, and Figure 8 and quantified by the alteration of the corresponding shape factors in Table 4 and Table 6. Additionally, stronger dissolution of Mg_2_Si is clearly recognizable in all alloys after homogenization at the higher temperature of 550 °C [6].

Besides an alteration of the primary phases, the homogenization treatment results in the formation of finely dispersed second phase particles [1]. As shown in Figure 5 and Figure 6 for the 500 °C homogenization or Figure 9 and Figure 10 for the 550 °C homogenization, precipitates on the scale of nano- up to micrometers can be observed. The influence of a homogenization time–temperature-cycle was already studied to some extent in [24,26,29,34,35]. The size, number density and composition of the thermodynamically stable phases varies with the alloying contents. In the present alloys, different stable dispersoid phases were found in the TEM measurements. Figure 11 highlights the formation of two different dispersoid types, Al_6_(Fe,Mn) and Al_15_(Fe,Mn)_3_Si_2_, in dependence on the local solute Si level.

In the absence of high concentrations of solute elements, the low Mn alloys LFe-LMn and HFe-LMn do not show distinct secondary phase formation with fractions only around 0.05–0.11 vol.% for the 500 °C homogenization and 0.05–0.22 vol.% for the 550 °C homogenization, respectively (Table 5 and Table 7). The differences for S-C and NR-C can be attributed to the increased solute Fe level for the higher cooling rates, resulting in increased driving forces for secondary phase precipitation. Concerning the size and aspect ratio of the dispersoids, the low Mn alloys do not depict clear trends.

With the increase in Mn content, the alloys show heavy precipitation of rod-shaped particles. As stated in [13], the 3D shape of the particles is rather plate-like than needle- or rod-shaped, and results after homogenization treatments at higher temperatures (550 °C). For lower temperatures also the formation of more spherical particles is mentioned in [13,36], which can be found to some extent in the micrographs Figure 6a and Figure 10a. With longer times and higher homogenization temperatures, the bigger (plate-like) dispersoids can grow at the expense of the small (spherical) ones. This trend is clearly observed for the HFe-HMn alloy in Figure 6d and Figure 10d and found in the rise of the average radius from 86 nm to 158 nm [24].

Furthermore, the alloys show considerable influence of the Fe alloying content on the secondary phase formation. In [36] the interchangeability of Fe and Mn in the dispersoids of different types is reported, which is also confirmed in the present study. With the starting precipitation of Mn containing dispersoids, the remaining solute Fe atoms will attach to these particles and likely build long, drawn-out second phase particles. While the LFe-HMn alloy shows medium-high volume fractions of Al_6_Mn type dispersoids, the higher Fe level in HFe-HMn, although usually not considered to be in solid solution to a large extent after casting, favors the formation of coarsened particles and raises the total volume fraction significantly. These considerations are highly affirmed since the behavior is more clearly found for the NR-C conditions. As an extremum, secondary phases of about 2.5 µm are found for the 550 °C homogenization of NR-C cast HFe-HMn alloy (Figure 10). Those big phases also affect the significance of the dispersoid volume fractions, as there are also large regions without obtainable particles in those samples, though particle containing images are evaluated.

The thermodynamic calculations in the present study are in very good agreement with the results of the experimental work. The general considerations of Mn and Fe solute content in non-equilibrium and equilibrium conditions are concordant to the precipitation behavior of secondary phase particles. Whereas the amounts of phases computed for the particular conditions do not totally agree with the experimental findings, they support current understanding of the trends of phase formation. Especially the for the high Fe containing alloys, the calculation underestimates the segregation of Fe in comparison to experimental data (e.g., HFe-LMn in Table 3 and Table 8). Difficulties arise for equilibrium conditions, since the calculation only displays data for the absolute volume fractions of the stated phases including primary and secondary phases. However, consideration of the different fractions from Table 6 and Table 7 repeatedly show the disagreement of the data mainly for high Fe containing alloys.

In contrast to the calculation of volume fractions, the thermodynamic calculation clearly agrees with the experimentally found types of primary and secondary phases, in particular for the non-equilibrium state. However, the HFe-HMn alloy in the S-C conditions show a distinct discrepancy as the calculated Al_15_(Fe,Mn)_3_Si_2_ phase is absent in the microstructure. The computed precipitation of both types of dispersoids, Al_6_(Fe,Mn) and Al_15_(Fe,Mn)_3_Si_2_, is proved (Figure 11) for the equilibrium conditions, although the verification of the given volume fractions of dispersoids goes beyond the scope of the experimental work. Finally, there are still difficulties in understanding the primary phase transformations tabulated in Table 8, since the experimental observations did not show major alterations of those intermetallics.

The final properties of the aluminum sheet after soft annealing are strongly influenced by the precipitation state of the intermetallics [1,11,24,35]. The fragmentation of primary phases during rolling (as shown in Figure 12 and Figure 13 for the different casting cooling conditions) will certainly affect the recrystallization during annealing. In the vicinity of larger phase-fragments (>1 µm), the mechanism of particle stimulated nucleation might occur [37], whereas smaller primary phase particles can also contribute to the Zener pinning pressure usually exerted by dispersoids [38].

In this context, the rod-shape of the dispersoids and their alignment with RD (Figure 14 and Figure 15) are crucial for the exerted pinning forces on grain boundaries growing in the different sample directions [38]. Comparing the dispersoids radii after homogenization and soft annealing, the significant reduction is attributed to a fragmentation of the particles during rolling. Since the probability is high that very small particles are not detected when evaluating the dispersoid fraction in the rolled and soft annealed condition, the decrease in the dispersoid volume fraction appears to be fairly plausible (compare Table 5 or Table 7 to Table 9 or Table 10).

The present alloys cover a wide range of type, volume fraction, size, and distribution of the primary and secondary phases in the matrix. With the shown band-wise arrangement of primary phases and the preferred orientation of smaller rod-shaped dispersoids, a considerable influence on the final sheet recrystallization is to be expected. These effects will be discussed in the subsequent paper (part II), including the discussion of the grain size, the grain morphology, and the texture of the alloys in the final sheet state.

## 5. Conclusions

The presented study investigates the evolution of primary and secondary phases in Al4.5Mg alloys with varied Fe and Mn levels as well as different casting and homogenization parameters at laboratory scaled production. The resulting microstructures are explained by micrographs, EDX phase characterization, and evaluated data on primary phase and dispersoid volume fractions. Furthermore, the experimentally observed occurrence and modification of primary and secondary phases are discussed in the light of thermodynamic calculations. Finally, the following conclusions can be drawn:

- High Fe/Mn ratios in the alloy contents favor the precipitation of Al_3_(Fe,Mn) primary phase in a characteristic needle-like shape. By increasing the Mn level, the primary phase coarsens and more likely forms Al_6_(Fe,Mn) or Al_15_(Fe,Mn)_3_Si_2_, depending on the dissolved Si solute content and the primary precipitation of Mg_2_Si, respectively.
- With near rapid cooling conditions in the casting process, the casting grain- and primary phase size are significantly reduced. The formation of different primary phase types in comparison to slow cooling conditions can be observed.
- The volume fractions of the primary phases considerably increase with the alloying contents, foremost by the Fe additions. The homogenization does not change the primary phases volume fractions nor compositions considerably.
- The homogenization heat treatment causes formation of secondary phases (Al_6_(Fe,Mn) or Al_15_(Fe,Mn)_3_Si_2_), especially in the high Mn containing alloys. The influence of the cooling rate during casting is marginally visible. Higher homogenization temperatures and higher Fe contents clearly result in coarsening of the plate-like dispersoids.
- Thermodynamic calculations highly affirm the experimental results. Besides the good conformity in the observed primary and secondary phase intermetallic types, the trends of the computed volume fractions accord with evaluated data from the micrographs.
- The multitude of primary and secondary phase states created by this experimental work highly impacts the final aluminum sheets microstructure and properties, which will be discussed in part two of the present study.

## Figures and Tables

**Table 1:** Chemical composition of the investigated alloys (wt.%).

Alloy	Mg	Si	Fe	Mn
LFe-LMn	4.51	0.12	0.10	0.22
LFe-HMn	4.40	0.12	0.11	0.94
HFe-LMn	4.48	0.11	0.40	0.22
HFe-HMn	4.56	0.11	0.39	1.04

**Table 2:** Fraction, shape, and type of the primary phases in S-C cast samples, evaluated by automated feature analysis.

	f_total_ [%]	f_bright_ [%]	f_dark_ [%]	Fe/Mn	SF	Al_3_(Fe,Mn)	Al_6_(Fe,Mn)	Al_15_(Fe,Mn)_3_Si_2_
LFe-LMn	0.90 ± 0.12	0.42 ± 0.24	0.48 ± 0.12	5.0 ± 1.0	1.78 ± 1.24	yes	no	no
LFe-HMn	1.48 ± 0.26	0.96 ± 0.33	0.52 ± 0.07	0.6 ± 0.4	3.06 ± 2.81	little	little	yes
HFe-LMn	1.46 ± 0.43	1.00 ± 0.34	0.46 ± 0.09	7.9 ± 2.4	2.08 ± 1.37	yes	no	no
HFe-HMn	4.09 ± 0.78	3.49 ± 0.93	0.60 ± 0.15	0.8 ± 0.5	3.37 ± 2.80	little	yes	no

f_total_, f_bright_, f_dark_: total area fraction of primary phases or area fractions of bright and dark phases respectively; Fe/Mn: Ratio of iron to manganese contents of the bright phase particles; SF: shape factor.

**Table 3:** Fraction, shape, and type of the primary phases in NR-C cast samples, evaluated by automated feature analysis.

	f_total_ [%]	f_bright_ [%]	f_dark_ [%]	Fe/Mn	SF	Al_3_(Fe,Mn)	Al_6_(Fe,Mn)	Al_15_(Fe,Mn)_3_Si_2_
LFe-LMn	0.83 ± 0.16	0.39 ± 0.02	0.44 ± 0.14	6.1 ± 2.5	1.57 ± 1.01	yes	no	no
LFe-HMn	1.23 ± 0.21	0.73 ± 0.14	0.50 ± 0.07	0.7 ± 0.3	1.54 ± 1.06	no	little	yes
HFe-LMn	2.24 ± 0.42	1.89 ± 0.29	0.35 ± 0.13	8.6 ± 3.5	2.30 ± 2.42	yes	no	no
HFe-HMn	2.31 ± 0.13	1.90 ± 0.18	0.41 ± 0.05	1.3 ± 0.3	2.56 ± 2.17	no	yes	yes

f_total_, f_bright_, f_dark_: total area fraction of primary phases or area fractions of bright and dark phases respectively; Fe/Mn: ratio of iron to manganese contents of the bright phase particles; SF: shape factor.

**Table 4:** Fraction, shape, and composition of the primary phases after homogenization at 500 °C (S-C and NR-C samples).

		f_total_ [%]	f_bright_ [%]	f_dark_ [%]	Fe/Mn	SF
S-C cast	LFe-LMn	0.71 ± 0.11	0.30 ± 0.03	0.40 ± 0.08	5.7 ± 2.0	2.10 ± 1.73
	LFe-HMn	1.16 ± 0.36	0.79 ± 0.33	0.37 ± 0.03	0.6 ± 0.3	2.80 ± 2.84
	HFe-LMn	1.22 ± 0.08	0.87 ± 0.27	0.35 ± 0.19	8.5 ± 1.0	1.93 ± 1.40
	HFe-HMn	2.67 ± 0.27	2.25 ± 0.20	0.42 ± 0.07	0.8 ± 0.2	2.40 ± 1.88
NR-C cast	LFe-LMn	0.74 ± 0.15	0.36 ± 0.12	0.38 ± 0.03	4.5 ± 1.7	1.88 ± 1.17
	LFe-HMn	0.91 ± 0.47	0.71 ± 0.37	0.20 ± 0.10	0.6 ± 0.2	1.51 ± 0.75
	HFe-LMn	1.53 ± 0.38	1.30 ± 0.30	0.33 ± 0.09	8.6 ± 2.7	2.00 ± 1.12
	HFe-HMn	2.82 ± 0.62	2.39 ± 0.52	0.42 ± 0.10	1.1 ± 0.3	2.27 ± 1.69

f_total_, f_bright_, f_dark_: total area fraction of primary phases or area fractions of bright and dark phases respectively; Fe/Mn: ratio of iron to manganese contents of the bright phase particles; SF: shape factor.

**Table 5:** Secondary phase morphology and volume fraction in S-C and NR-C cast samples homogenized at 500 °C.

		f_D_ [vol.%]	r [nm]	AR
S-C cast	LFe-LMn	0.021	59	0.65
	LFe-HMn	0.756	87	0.57
	HFe-LMn	0.024	74	0.55
	HFe-HMn	1.440	84	0.57
NR-C cast	LFe-LMn	0.061	87	0.57
	LFe-HMn	0.620	71	0.58
	HFe-LMn	0.117	74	0.65
	HFe-HMn	2.341	86	0.57

f_D_: volume fraction of dispersoids; r: average radius of the dispersoid particles; AR: aspect ratio of the dispersoids.

**Table 6:** Fraction, shape, and composition of the primary phases after homogenization at 550 °C (S-C and NR-C samples).

		f_total_ [%]	f_bright_ [%]	f_dark_ [%]	Fe/Mn	SF
S-C cast	LFe-LMn	0.51 ± 0.14	0.28 ± 0.05	0.23 ± 0.09	4.3 ± 1.8	1.83 ± 1.38
	LFe-HMn	1.06 ± 0.05	0.90 ± 0.01	0.16 ± 0.04	0.3 ± 0.2	1.84 ± 1.57
	HFe-LMn	1.29 ± 0.21	1.00 ± 0.14	0.29 ± 0.07	7.9 ± 1.3	1.71 ± 1.46
	HFe-HMn	3.54 ± 0.32	3.22 ± 0.20	0.32 ± 0.12	0.8 ± 0.2	2.80 ± 2.68
NR-C cast	LFe-LMn	0.73 ± 0.20	0.43 ± 0.18	0.30 ± 0.02	3.1 ± 1.8	1.45 ± 0.67
	LFe-HMn	0.95 ± 0.05	0.86 ± 0.09	0.09 ± 0.04	0.3 ± 0.2	1.42 ± 0.73
	HFe-LMn	1.63 ± 0.50	1.37 ± 0.41	0.26 ± 0.10	7.1 ± 2.9	1.81 ± 1.17
	HFe-HMn	3.16 ± 0.68	2.90 ± 0.58	0.27 ± 0.11	0.5 ± 0.2	1.76 ± 1.15

f_total_, f_bright_, f_dark_: total area fraction of primary phases or area fractions of bright and dark phases respectively; Fe/Mn: ratio of iron to manganese contents of the bright phase particles; SF: shape factor.

**Table 7:** Secondary phase morphology and volume fraction in S-C and NR-C cast samples homogenized at 550 °C.

		f_D_ [vol.%]	r [nm]	AR
S-C cast	LFe-LMn	0.047	144	0.56
	LFe-HMn	1.036	124	0.55
	HFe-LMn	0.108	74	0.58
	HFe-HMn	2.252	123	0.57
NR-C cast	LFe-LMn	0.062	108	0.60
	LFe-HMn	0.741	105	0.54
	HFe-LMn	0.217	126	0.59
	HFe-HMn	2.293	158	0.51

f_D_: volume fraction of dispersoids; r: average radius of the dispersoid particles; AR: aspect ratio of the dispersoids.

**Table 8:** Results of the thermodynamic calculations using Pandat software for the alloys investigated.

	Fe in Fcc ×10^−3^ [wt.%] Nonequi./Equi.	Mn in Fcc [wt.%] Nonequi./Equi.	Al_13_(Fe,Mn)_4_ [vol.%] Nonequi./Equi.	Al_15_(Fe,Mn)_3_Si_2_ [vol.%] Nonequi./Equi.	Al_6_(Fe,Mn) [vol.%] Nonequi./Equi.
LFe-LMn	7.6/4.7	0.195/0.092	0.20/-	-	-/0.54
LFe-HMn	7.0/1.0	0.700/0.257	0.08/-	0.31/0.90	0.87/1.57
HFe-LMn	17.0/6.7	0.165/0.066	0.77/0.56	-/0.51	-
HFe-HMn	15.0/5.2	0.651/0.233	0.67/0.20	0.40/1.62	0.78/1.28

Fe,Mn in fcc: solute content of iron and manganese in the aluminum matrix; nonequi./equi.: calculated contents for nonequilibrium (Scheil) conditions/calculated contents for equilibrium conditions at 550 °C.

**Table 9:** Secondary phase morphology and volume fraction in S-C cast, processed and soft annealed sample states.

Sample State		Alloy	f_D_ [vol.%]	r [nm]	AR
Homogenization	CRD				
500 °C	35%	LFe-LMn	0.01	78	0.60
		LFe-HMn	0.96	81	0.50
		HFe-LMn	0.01	45	0.57
		HFe-HMn	0.64	62	0.55
	63%	LFe-LMn	0.04	43	0.65
		LFe-HMn	0.75	61	0.51
		HFe-LMn	0.02	39	0.60
		HFe-HMn	0.63	58	0.55
550 °C	35%	LFe-LMn	0.03	75	0.55
		LFe-HMn	0.64	82	0.53
		HFe-LMn	0.01	72	0.59
		HFe-HMn	1.35	78	0.52
	63%	LFe-LMn	0.01	72	0.66
		LFe-HMn	1.27	91	0.51
		HFe-LMn	0.03	45	0.65
		HFe-HMn	1.63	92	0.47

f_D_: volume fraction of dispersoids; r: average radius of the dispersoid particles; AR: aspect ratio of the dispersoids.

**Table 10:** Secondary phase morphology and volume fraction in NR-C cast, processed and soft annealed sample states.

Sample State		Alloy	f_D_ [vol.%]	r [nm]	AR
Homogenization	CRD				
500 °C	35%	LFe-LMn	0.02	54	0.68
		LFe-HMn	1.11	74	0.48
		HFe-LMn	0.05	62	0.49
		HFe-HMn	0.90	61	0.49
	63%	LFe-LMn	0.01	50	0.65
		LFe-HMn	1.01	73	0.47
		HFe-LMn	0.08	67	0.51
		HFe-HMn	1.07	63	0.48
550 °C	35%	LFe-LMn	0.01	58	0.65
		LFe-HMn	0.82	71	0.52
		HFe-LMn	0.03	77	0.58
		HFe-HMn	1.20	88	0.50
	63%	LFe-LMn	0.04	65	0.62
		LFe-HMn	0.58	81	0.47
		HFe-LMn	0.06	76	0.61
		HFe-HMn	1.00	79	0.52

f_D_: volume fraction of dispersoids; r: average radius of the dispersoid particles; AR: aspect ratio of the dispersoids.

## Appendix A

**Table A1:** Summarized data on primary phases of various sample states.

Sample State	Alloy	f_bright_ [%]	Fe/Mn	SF	Sample State	f_bright_ [%]	Fe/Mn	SF
S-C	LFe-LMn	0.42 ± 0.24	5.0 ± 1.0	1.78 ± 1.24	NR-C	0.39 ± 0.02	6.1 ± 2.5	1.57 ± 1.01
	LFe-HMn	0.96 ± 0.33	0.6 ± 0.4	3.06 ± 2.81		0.73 ± 0.14	0.7 ± 0.3	1.54 ± 1.06
	HFe-LMn	1.00 ± 0.34	7.9 ± 2.4	2.08 ± 1.37		1.89 ± 0.29	8.6 ± 3.5	2.30 ± 2.42
	HFe-HMn	3.49 ± 0.93	0.8 ± 0.5	3.37 ± 2.80		1.90 ± 0.18	1.3 ± 0.3	2.56 ± 2.17
S-C, 500 °C	LFe-LMn	0.30 ± 0.03	5.7 ± 2.0	2.10 ± 1.73	NR-C, 500 °C	0.36 ± 0.12	4.5 ± 1.7	1.88 ± 1.17
	LFe-HMn	0.79 ± 0.33	0.6 ± 0.3	2.80 ± 2.84		0.71 ± 0.37	0.6 ± 0.2	1.51 ± 0.75
	HFe-LMn	0.87 ± 0.27	8.5 ± 1.0	1.93 ± 1.40		1.30 ± 0.30	8.6 ± 2.7	2.00 ± 1.12
	HFe-HMn	2.25 ± 0.20	0.8 ± 0.2	2.40 ± 1.88		2.39 ± 0.52	1.1 ± 0.3	2.27 ± 1.69
S-C, 550 °C	LFe-LMn	0.28 ± 0.05	4.3 ± 1.8	1.83 ± 1.38	NR-C, 550 °C	0.43 ± 0.18	3.1 ± 1.8	1.45 ± 0.67
	LFe-HMn	0.90 ± 0.01	0.3 ± 0.2	1.84 ± 1.57		0.86 ± 0.09	0.3 ± 0.2	1.42 ± 0.73
	HFe-LMn	1.00 ± 0.14	7.9 ± 1.3	1.71 ± 1.46		1.37 ± 0.41	7.1 ± 2.9	1.81 ± 1.17
	HFe-HMn	3.22 ± 0.20	0.8 ± 0.2	2.80 ± 2.68		2.90 ± 0.58	0.5 ± 0.2	1.76 ± 1.15

Sample state: cast and homogenization conditions of the samples; f_bright_: area fraction of bright phases; Fe/Mn: ratio of iron to manganese contents of the bright phase particles; SF: shape factor.

**Table A2:** Summarized data on secondary phases (dispersoids) of various sample states.

Sample State	Alloy	f_D_ [vol.%]	r [nm]	AR	Sample State	f_D_ [vol.%]	r [nm]	AR
S-C, 500 °C	LFe-LMn	0.021	59	0.65	NR-C, 500 °C	0.061	87	0.57
	LFe-HMn	0.756	87	0.57		0.620	71	0.58
	HFe-LMn	0.024	74	0.55		0.117	74	0.65
	HFe-HMn	1.440	84	0.57		2.341	86	0.57
S-C, 550 °C	LFe-LMn	0.047	144	0.56	NR-C, 550 °C	0.062	108	0.60
	LFe-HMn	1.036	124	0.55		0.741	105	0.54
	HFe-LMn	0.108	74	0.58		0.217	126	0.59
	HFe-HMn	2.252	123	0.57		2.293	158	0.51

f_D_: volume fraction of dispersoids; r: average radius of the dispersoid particles; AR: aspect ratio of the dispersoids.
